# Biochar Composite with Enhanced Performance Prepared Through Microbial Modification for Water Pollutant Removal

**DOI:** 10.3390/ijms252111732

**Published:** 2024-10-31

**Authors:** Bolun Zhang, Ruqi Li, Yangyang Zheng, Siji Chen, Yingjie Su, Wei Zhou, Qi Sui, Dadong Liang

**Affiliations:** 1 College of Life Sciences, Jilin Agricultural University, Changchun 130118, China; aa921526347@163.com (B.Z.); 14752267617@163.com (R.L.); z08130214@163.com (Y.Z.); zhouwei6423@126.com (W.Z.); suiqi0125@126.com (Q.S.); liangdadong@jlau.edu.cn (D.L.); 2 Key Laboratory of Straw Comprehensive Utilization and Black Soil Conservation, Ministry of Education, Jilin Agricultural University, Changchun 130118, China

**Keywords:** biochar, bio-modification, fungal, contamination, adsorption

## Abstract

This study developed mycelial biochar composites, BQH-AN and BQH-MV, with stable physicochemical properties and significantly improved adsorption capabilities through microbial modification. The results showed that the specific surface area and porosity of BQH-AN (3547.47 m^2^ g^−1^ and 2.37 cm^3^ g^−1^) and BQH-MV (3205.59 m^2^ g^−1^ and 2.46 cm^3^ g^−1^) were significantly higher than those of biochar BQH (2641.31 m^2^ g^−1^ and 1.81 cm^3^ g^−1^), which was produced without microbial treatment. In adsorption experiments using rhodamine B (RhB), tetracycline hydrochloride (TC), and Cr (VI), BQH-AN showed maximum adsorption capacities of 1450.79 mg g^−1^ for RhB, 1608.43 mg g^−1^ for TC, and 744.15 mg g^−1^ for Cr(VI). BQH-MV showed similarly strong performance, with 1329.85 mg g^−1^ for RhB, 1526.46 mg g^−1^ for TC, and 752.27 mg g^−1^ for Cr(VI). These values were not only higher than those of BQH but also outperformed most other biochar adsorbents. Additionally, after five reuse cycles, the pollutant removal efficiency of the mycelial biochar composites remained above 69%, demonstrating excellent regenerative ability. This study not only produced biochar with superior adsorption properties but also highlighted microbial modification as an effective way to enhance lignocellulosic biochar performance, paving the way for further biomass development.

## 1. Introduction

With the rapid acceleration of industrialization worldwide, the discharge of various pollutants is increasingly degrading water environments [1]. Pollutants such as dyes, antibiotics, and heavy metals pose serious threats to human health and ecosystems [2]. In response to the growing need for clean drinking water, various water treatment methods have been developed, including biodegradation, advanced oxidation processes, flocculation, membrane filtration, and adsorption [3,4,5]. Among these, adsorption is considered one of the most effective methods for environmental water remediation due to its versatile material design, ease of operation, and cost-effectiveness [6,7].

Adsorbents are considered to be one of the most important factors affecting the adsorption efficiency, so the development of efficient, environmentally friendly, and affordable adsorbents is crucial for the treatment of water pollution. Biochar has emerged as a promising adsorbent because of its abundant availability, porous structure, and the presence of unsaturated functional groups on its surface. It is commonly produced from feedstocks such as agricultural waste, animal manure, sewage sludge, and food waste [8,9]. Agricultural by-products such as straw, rice hulls, quinoa hulls, and other woody biomass are frequently used to produce biochar due to their renewability and wide availability [10,11]. This approach to utilizing agricultural waste plays an essential role in addressing energy shortages and promoting sustainable agricultural practices. However, traditionally produced biochar has limitations in adsorption performance, including insufficient pollutant capture and low removal efficiency [12,13]. To enhance biochar performance, several modification techniques have been applied, such as surface modification [14], physical modification [15], chemical modification [16,17], and oxidation using agents such as H_2_O_2_ [18]. Unlike more costly or environmentally harmful alternatives, bio-regulation methods using microorganisms, such as fungi, offer a gentle and cost-effective solution that aligns with green sustainable practices [19,20]. For instance, Nie et al. developed a novel biochar by pyrolyzing *Aspergillus aryabhattai* (AOMA) floc, which was then used to remediate tetracycline-contaminated water [21]. Similarly, Huang et al. identified a bacterium (*Bacillus aryabhattai*) with high efficiency in removing arsenic (As) and cadmium (Cd) and developed a biochar composite integrated with *Bacillus aryabhattai* through physical adsorption and sodium alginate embedding. This biochar composite significantly reduced the exchangeable As and Cd fractions in co-contaminated soil [22]. However, up to date, it is still a challenge to effectively implement bio-modification to improve the efficiency of functionalized materials further to deal with environmental pollution treatment.

Agricultural waste, which comprises a large amount of by-products from agricultural production and the processing of agricultural products, is mainly composed of lignocellulose, which is often considered a high-quality carbon source precursor for the development of biochar. Lignocellulosic biomass is primarily composed of lignin, cellulose, and hemicellulose, which are cross-linked to form a compact structure [23]. This structure restricts the development of the biochar’s pore network during the activation process [24]. However, studies have shown that fungi like *Aspergillus niger*, *Trichoderma reesei*, and *Myrothecium verrucaria* can secrete enzymes such as cellulase, hemicellulase, and ligninase during growth [25,26,27]. These enzymes effectively break down the lignocellulosic structure, creating a loosely arranged biochar precursor. This bio-regulation process, when combined with char activation, significantly enhances the specific surface area and pore volume of the resulting biochar [28]. While some studies remove fungal mycelium from the material to improve purity, this separation increases energy consumption and experimental costs. Research has shown that fungal mycelium, which forms a three-dimensional network structure during growth, can serve as an efficient composite template [29,30]. This structural transformation provides a larger specific surface area, and when co-pyrolyzed with woody fibrous biomass, it may introduce additional unsaturated functional groups, further improving biochar adsorption performance. The scarcity of reports on whether utilizing fungal mycelium’s bio-modification function can effectively improve biochar properties encourages us to explore this interesting topic.

In this study, we investigated the feasibility of using bio-regulation methods to enhance the degradative properties of fungi for preparing high-specific-surface-area biochar. Fungal composite biochar was produced via the co-pyrolysis of fungi-treated biomass, using quinoa husk as the precursor. *Aspergillus niger* (AN) and *Myrothecium verrucaria* (MV) were selected as the pretreatment fungi. Rhodamine B (RhB), tetracycline hydrochloride (TC), and Cr(VI) were used as model pollutants. A series of adsorption experiments—including adsorption kinetics, isotherms, thermodynamics, and fixed-bed studies—were conducted to elucidate the mechanisms behind the pollutants’ adsorption onto the fungal composite biochar. The study aimed to assess the effectiveness of the bio-regulation approach in enhancing biochar adsorption performance by comparing the original biochar with the fungal composite biochar.

## 2. Results and Discussion

### 2.1. Biochar Preparation Using Bio-Regulation Method

The flow chart for preparing mycelial composite biochar through the bio-regulation method is shown in Figure S1. During the solid fermentation process, fungal spores grow and develop into mycelium in the quinoa husk (QH) solid medium. These mycelia secrete bioenzymes that degrade the wood fiber structure. This biodegradation weakens the tightly bound wood fibers in the QH, making the material’s structure more loose [25,26]. Additionally, as the mycelium grows, it forms a three-dimensional network structure that wraps around the QH, creating a mycelium/QH complex. These structural changes may work synergistically in the subsequent biochar preparation process, enhancing the pore volume and specific surface area of biochar and improving the adsorption capacity.

The bio-regulation conditions for preparing mycelial composites were optimized based on their pollutant adsorption capacity. Key factors such as fermentation time, temperature, and the amount of fungal inoculum were considered (Figure S2). The results showed that the highest adsorption capacity for pollutants by BQH-AN and BQH-MV was achieved with a solid fermentation time of 21 days, a 3% inoculum, and solid–liquid ratios of 1:2 and 1:1.5. These were determined to be the optimal conditions for bio-regulation.

### 2.2. Characterization Results

**Figure 1:**
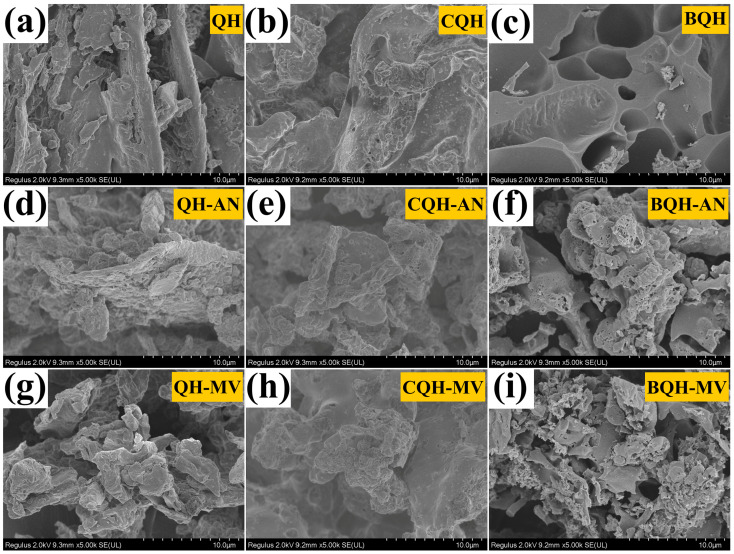
The SEM images of (**a**) QH, (**b**) CQH, (**c**) BQH, (**d**) QH-AN, (**e**) CQH-AN, (**f**) BQH-AN, (**g**) QH-MV, (**h**) CQH-MV, and (**i**) BQH-MV.

The structure and elemental composition of the materials were analyzed using SEM and EDS (Figure 1 and Figure S3). Compared to the QH, the bio-modified QH-AN and QH-MV exhibited rougher surfaces and more fragmented structures, likely due to the degradation of the wood fiber structure by the bioenzymes secreted by fungi during solid fermentation [31]. After carbonization, the QH had a smooth surface, whereas CQH-AN displayed a richer pore structure. Additionally, filamentous fungi were observed covering the surface of CQH-MV, indicating successful mycelium complexation. These results suggest that the benefits introduced by bio-regulation were preserved through the carbonization process. After the activation reaction, the pore structures of the bio-regulated biochar were more developed, likely due to a synergistic effect between the activator and the structure disruption of the QH’s wood fibers caused by bio-regulation [31,32,33]. This structural modification increases the specific surface area and pore volume, enhancing the material’s effectiveness in treating water pollutants.

EDS revealed that BQH-AN and BQH-MV contained a higher percentage of nitrogen compared to the untreated biochar (Table S1). This increase in nitrogen content likely resulted from the co-pyrolysis of the bio-regulated mycelial complexes during biochar preparation. Nitrogen-containing functional groups, such as pyrrole nitrogen, pyridine nitrogen, and graphite nitrogen, play a significant role in pollutant adsorption [34,35].

Thermal characterization of the three raw material samples was performed using TGA and DTG (Figure S4). The results showed an initial mass loss occurring below 200 °C, primarily due to the removal of moisture and volatile gasses. The second stage of mass loss (200–500 °C) was attributed to the pyrolysis of lignocellulose components and the decomposition of proteins and other organic substances. The degradation temperatures were 220–325 °C for hemicellulose, 315–400 °C for cellulose, 250–500 °C for lignin, and 210–400 °C for proteins and carbohydrates [36,37]. The third stage of mass loss, observed above 500 °C, was associated with the production of CO and CO_2_ from the high-temperature decomposition of carbonaceous residues. Based on the TGA and DTG results, 600 °C was selected as the carbonization temperature, with the mass residues of the three samples being 34%, 35%, and 39%, respectively. These findings indicate that the thermal stability of mycelial composite materials prepared via bio-regulation was enhanced.

The FT-IR spectroscopy results (Figure S5) revealed that the broad peaks around 3400 cm^−1^ were related to the stretching vibrations of -OH groups. These peaks became more defined after charring and activation, indicating that the biochar conversion process involved the transformation of oxygen-containing functional groups [38,39]. Small peaks near 2920 cm^−1^ and 2850 cm^−1^ corresponded to C-H stretching vibrations in cellulose and hemicellulose, while peaks between 1600 and 1650 cm^−1^ represented C=C or C=O stretching vibrations in carbonyl groups. The peak at 1420 cm^−1^ was attributed to the asymmetric bending of CH_2_. During pyrolysis at high temperatures, the aromatic and aliphatic C-H bonds were thermally unstable and largely disappeared [39,40]. The peak near 1320 cm^−1^ was associated with carboxylic acid from lignin, while the peak near 1052 cm^−1^ indicated C-O-C bonds in cellulose and hemicellulose [40]. These carbon- and oxygen-containing functional groups may provide new adsorption sites, contributing positively to pollutant removal.

The crystalline structure and degree of carbon defects in the samples were analyzed using XRD and Raman spectroscopy (Figure S5). XRD results showed broad diffraction peaks around 23° and 43°, corresponding to the (002) and (100) crystalline planes of carbon, respectively, confirming the presence of both amorphous and graphitic carbon structures [41]. Raman spectroscopy revealed characteristic peaks at 1345 cm^−1^ and 1587 cm^−1^, corresponding to the defective graphitic structure or disordered carbon (D band) and the in-plane vibration of sp^2^ carbon atoms (G band) [42]. The intensity ratio of the D band to the G band (I_D_/I_G_) reflects the degree of defects in the carbon material. After activation, the I_D_/I_G_ of the samples increased (CQH: 3.2, CQH-AN: 3.2, CQH-AN: 3.2, CQH-AN: 2.1, CQH-AN: 2.98, CQH-MV: 2.35, BQH: 3.88, BQH-AN: 3.44, and BQH-MV: 2.98), suggesting that the relative content of amorphous carbon in the biochar rose, which may enhance pollutant removal during the adsorption process.

The chemical composition of the sample surfaces was analyzed using XPS spectroscopy (Figure S6 and Table S2). The results indicated that the relative nitrogen (N) content of QH-AN and QH-MV was higher than that of the original QH after bio-regulation, suggesting that mycelium involvement increased the N content in the composites. After carbonization and activation, the N contents of BQH-AN and BQH-MV remained higher than that of BQH, indicating that mycelium co-pyrolysis successfully endowed the composite biochar with increased nitrogen levels. However, the relative O and N contents decreased after carbonization and activation, while the relative C content increased, likely due to the pyrolysis process, which removed some O and N in the form of volatile gasses. The C1s, O1s, and N1s spectra of the samples (Figures S7 and S8) showed characteristic peaks at 531.8 eV, 533.0 eV, and 534.1 eV, corresponding to C-C/C=C/CH, C-OH, and C=O, respectively. Peaks at 284.8 eV, 285.5 eV, and 288.9 eV represented C-O/C=O, C-O-C, and -OH, respectively. In general, the carbon- and oxygen-containing functional groups in biochar act as electron donors and play an active role in pollutant adsorption [43,44].

The N1s band of the sample was deconvoluted into three characteristic peaks, namely pyridine nitrogen (397.8 eV), pyrrole nitrogen (399.1 eV), and graphitic nitrogen (400.2 eV). In the pristine carbon precursors, nitrogen existed mainly in the form of pyrrole and pyridine nitrogen. After carbon activation, some nitrogen was lost due to high-temperature pyrolysis, while the remaining nitrogen formed a graphitic structure with carbon [45]. Pyridine and pyrrole nitrogen can interact with pollutants through Lewis acid–base interactions, while graphitic nitrogen primarily enhances pollutant removal via π–π interactions and electron transfer [46]. The abundant surface functional groups in BQH-AN and BQH-MV provided more active sites, playing a key role in removing pollutants from water.

**Figure 2:**
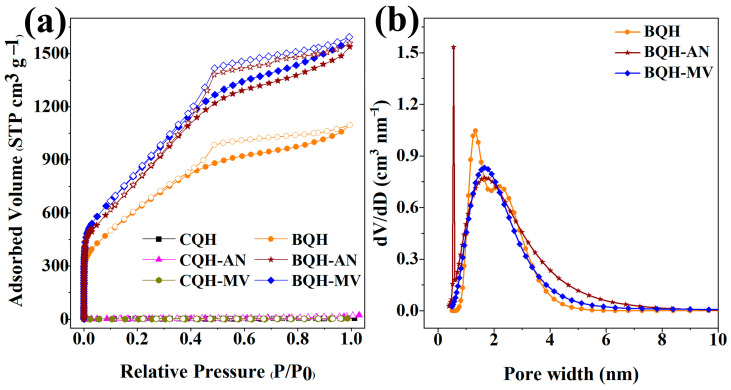
(**a**) N_2_ adsorption–desorption isotherms of CQH, CQH-AN, CQH-MV, BQH, BQH-AN, and BQH-MV; (**b**) pore size distribution curves of BQH, BQH-AN, and BQH-MV.

To evaluate the pore structure and surface properties of the biochar samples, the samples were tested for N_2_ adsorption–desorption isotherms and pore size distribution (Figure 2 and Table S3). The results showed that CQH-AN and CQH-MV had a significantly higher specific surface area and pore volume compared to CQH. However, their relatively lower specific surface area and pore volume indicated the need for further activation to enhance adsorption performance.

The activation process aimed to develop a more complex hierarchical pore structure using a mixture of sodium hydroxide and potassium hydroxide as activators due to the differing sizes of their alkali ions. The use of mixed bases helped maintain a stable chemical equilibrium, reducing excessive base consumption during the reaction and thereby improving activation efficiency and homogeneity. During pyrolysis, the mixed-base activator formed solvated complexes with water in the carbon precursor, containing cations (Na^+^ and K^+^) [36]. These positively charged complexes contributed to the pore structure by inserting or migrating into the carbon material’s framework, which was later removed using hydrochloric acid.

BQH, BQH-AN, and BQH-MV exhibited typical H1-type isotherms and H4-type hysteresis loops, indicating the presence of a hierarchical pore structure. The pore size distribution data demonstrated both microporous and mesoporous structures, which not only provided diffusion channels but also a large number of adsorption sites, playing a key role in pollutant adsorption. Specifically, BQH-AN and BQH-MV had significantly higher specific surface areas (3547.47 m^2^ g^−1^ and 2.37 cm^3^ g^−1^; 3205.59 m^2^ g^−1^ and 2.46 cm^3^ g^−1^) and pore volume compared to the original biochar (2641.31 m^2^ g^−1^ and 1.81 cm^3^ g^−1^). The alkali-to-carbon ratio of the mycelial composite biochar (1.5:1.5:1) was lower than that of BQH (2:2:1), likely due to the bio-regulation process, which improved interaction with the activator and reduced alkali consumption. The bio-regulation process, facilitated by mycelium doping, appeared to destroy the carbon precursor’s structure, promoting better interaction with the activator. The mycelium’s unique three-dimensional network structure likely contributed to the larger specific surface area and pore volume compared to the QH. This bio-regulation method improved activation efficiency and reduced activator consumption through a synergistic interaction with the activator. Additionally, it enhanced the specific surface area and pore volume by utilizing the mycelium’s distinct laminated structure. It is important to note that while the specific surface area of biochar includes both internal and external regions, the adsorption properties primarily depend on the internal surface formed by the pore structure [47]. Therefore, the hierarchical pore structure and high pore volume of BQH-AN and BQH-MV contributed to a larger specific surface area and more adsorption sites, significantly improving their adsorption capabilities.

### 2.3. Adsorption Kinetics Results

The study investigated the relationship between adsorption time and adsorption amount through adsorption kinetics [48,49]. For RhB and TC adsorption, BQH, BQH-AN, and BQH-MV exhibited a rapid adsorption phase within the first 10 min, followed by a gradual decrease in the adsorption rate until equilibrium was reached. Similarly, the adsorption of Cr(VI) displayed a fast adsorption phase within the first 20 min. During this rapid phase, pollutant molecules bind to a large number of unsaturated active sites, resulting in a high adsorption rate. As these active sites become occupied, the rate of adsorption slows until equilibrium is attained. In the fast adsorption phase, BQH, BQH-AN, and BQH-MV exhibited adsorption capacities of 56–59%, 86–91%, and 83–89% of the equilibrium capacity for RhB and 78–79%, 87–89%, and 87–89% for TC. For Cr(VI), the adsorption capacities were 63–64%, 85–87%, and 86–87%. These results suggest that BQH-AN and BQH-MV demonstrated superior rapid adsorption performance, likely due to their enhanced hierarchical pore structure and larger specific surface area.

**Figure 3:**
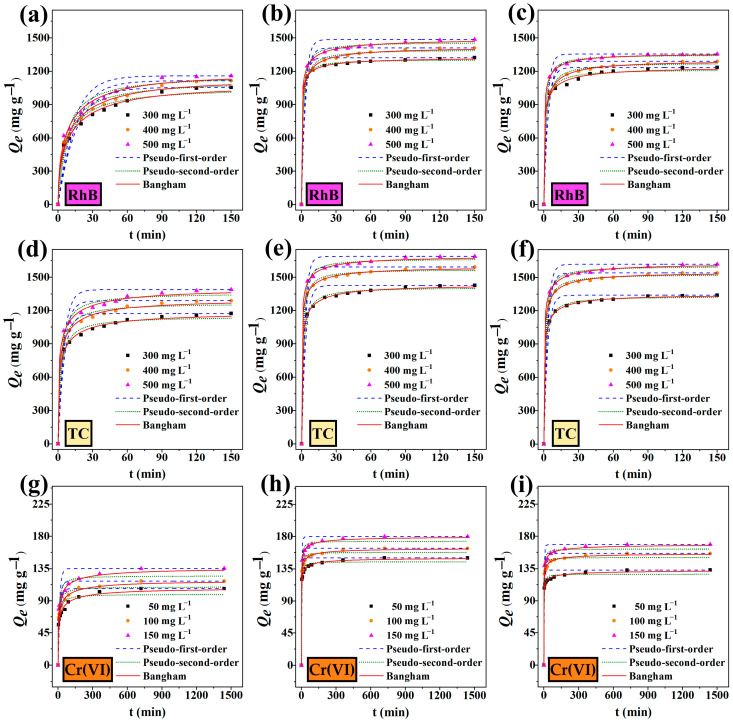
The pseudo first-order kinetic plots, the pseudo second-order kinetic plots, and the Bangham kinetic model plots of (**a**) BQH, (**b**) BQH-AN, and (**c**) BQH-MV for RhB; (**d**) BQH, (**e**) BQH-AN, and (**f**) BQH-MV for TC; and (**h**) BQH, (**i**) BQH-AN, and (**g**) BQH-MV for Cr(VI) at 303 K.

Adsorption data were analyzed using four kinetic models, which were the pseudo first-order, pseudo second-order, intraparticle diffusion, and Bangham models; these were used to explore potential adsorption mechanisms (Figure 3 and Figure S9 and Tables S4–S6). The pseudo second-order kinetic models for RhB, TC, and Cr(VI) adsorption exhibited high correlation coefficients (*R*^2^), indicating that chemical reactions, such as electron transfer or sharing, influenced the adsorption rate, forming chemisorption bonds. The Bangham model had the highest *R*^2^ values (all above 0.99), suggesting that both chemical adsorption and pore diffusion were involved in the adsorption process [36]. Additionally, lower SD and BIC values further validated the model’s suitability.

### 2.4. Adsorption Isotherms Results

**Figure 4:**
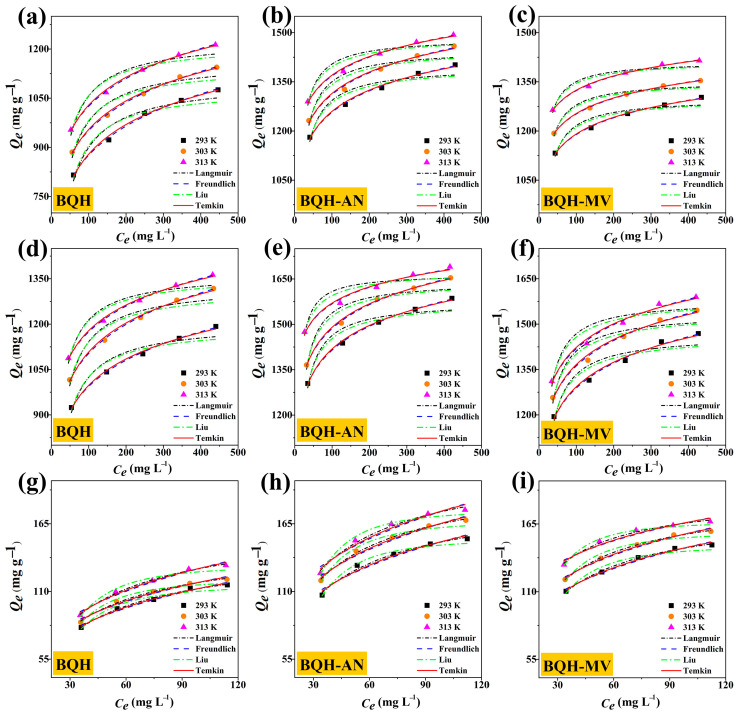
Adsorption isotherms of (**a**) BQH, (**b**) BQH-AN, and (**c**) BQH-MV for RhB; (**d**) BQH, (**e**) BQH-AN, and (**f**) BQH-MV for TC; and (**g**) BQH, (**h**) BQH-AN, and (**i**) BQH-MV for Cr(VI) at 303 K.

The Langmuir, Freundlich, Temkin, and Liu adsorption isotherm models were applied to describe the adsorption characteristics of the adsorbent and the substance transfer during the adsorption process. For RhB and TC adsorption (Figure 4 and Tables S7–S9), the Freundlich model exhibited a high *R*^2^ value (>0.98), suggesting multilayer adsorption on heterogeneous surfaces. Additionally, the *n_f_* value was greater than one, indicating a strong affinity of the adsorbent for the substances being adsorbed [29,30]. In contrast, for Cr(VI) adsorption, the Langmuir model showed a higher correlation coefficient (*R*^2^ > 0.99), implying that the adsorption process primarily involves monolayer adsorption at the active sites of the material. Furthermore, the adsorption of all three pollutants (RhB, TC, and Cr(VI)) fit the Temkin model well (*R*^2^ > 0.98), indicating that the process likely involves surface adsorption through interactions between the biochar and the pollutants, incorporating both chemisorption and physisorption mechanisms.

### 2.5. Adsorption Thermodynamics

**Figure 5:**
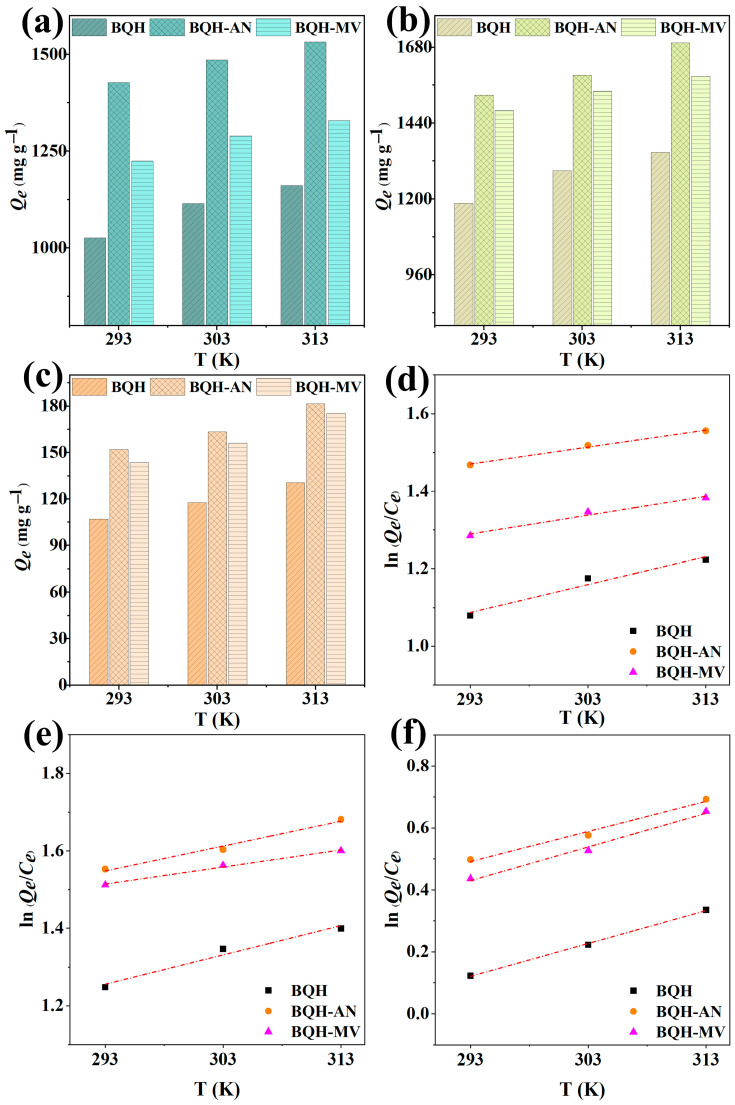
Effects of different temperatures on the adsorption capacities of BQH, BQH-AN, and BQH-MV for (**a**) RhB, (**b**) TC, and (**c**) Cr(VI); plot of ln(*Q_e_*/*C_e_*) versus T of BQH, BQH-AN, and BQH-MV for the adsorption of (**d**) RhB, (**e**) TC, and (**f**) Cr(VI).

The energy changes and thermodynamic properties (Figure 5 and Tables S10–S12) related to the adsorption process were analyzed using thermodynamic experiments and parametric calculations. Negative values of Δ*G* indicate that the adsorption of RhB, TC, and Cr(VI) by the samples occurs spontaneously. Typically, when Δ*H* exceeds 80 kJ·mol^−1^, it highlights significant heat release or absorption, pointing to chemisorption as the dominant mechanism. In contrast, physical adsorption, characterized by weaker interactions and the absence of new chemical bond formations, generally occurs when Δ*H* is less than 20 kJ·mol^−1^. The positive Δ*H* values observed in this study (23.98–35.22 kJ mol^−1^) suggest that the adsorption process is endothermic and governed by both physical and chemical adsorption mechanisms [50,51]. Additionally, the positive Δ*S* values suggest increased disorder at the solid–liquid interface, favoring adsorption.

### 2.6. Effect of pH

**Figure 6:**
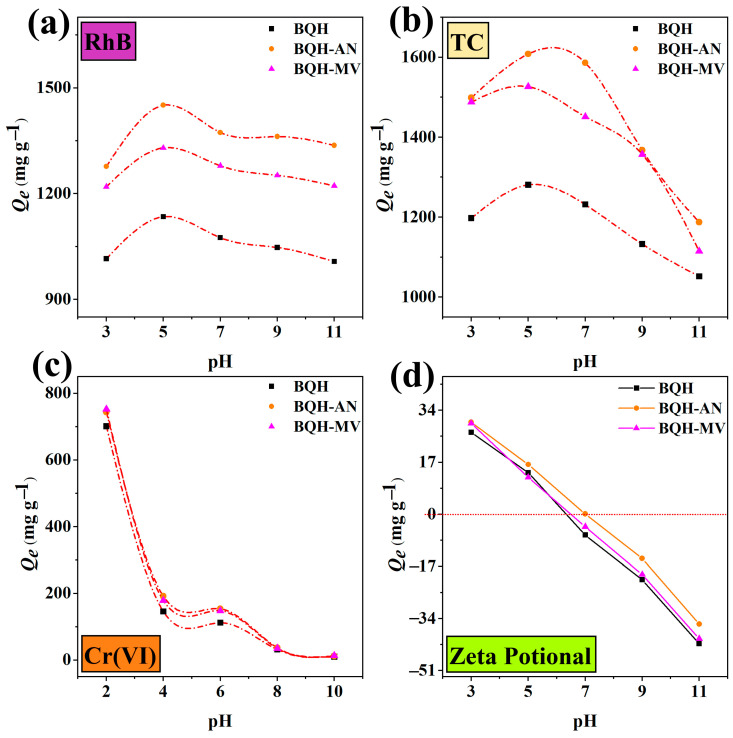
Effect of pH on the adsorption of (**a**) RhB, (**b**) TC, and (**c**) Cr(VI); (**d**) the zeta potential of BQH, BQH-AN, and BQH-MV at 303 K.

The pH significantly influences the interaction between the adsorbent and the adsorbed substance, playing a critical role in the adsorption process (Figure 6). The pH at the point of zero charge (pH_pzc_) for BQH, BQH-AN, and BQH-MV was found to be 6.28, 6.54, and 6.95, respectively. When the pH is below the pH_pzc_, the surface of the samples is positively charged, whereas it becomes negatively charged when the pH exceeds the pH_pzc_.

At pH levels below three, RhB primarily exists as RhB^+^, experiencing electrostatic repulsion from the positively charged surface of the samples, thereby reducing adsorption efficiency. However, at pH 5, RhB^−^ in the solution interacts electrostatically with the positively charged surfaces of BQH, BQH-AN, and BQH-MV, reaching maximum adsorption capacities of 1134.78, 1450.79, and 1329.85 mg g^−1^, respectively. Beyond pH 5, amphiphilic RhB molecules form dimers, hindering adsorption due to their large molecular size [52]. When the pH surpasses the pHpzc, the negatively charged adsorbent surface repels RhB^−^, and OH^−^ ions compete with the adsorbent for RhB molecules, reducing the adsorption capacity.

For TC, at pH values below 3.3, TC exists primarily as TC^+^, leading to electrostatic repulsion from the positively charged adsorbents. As the pH increases to five, TC exists mainly as TC^0^, resulting in maximum adsorption capacities for BQH, BQH-AN, and BQH-MV of 1280.88, 1608.43, and 1526.46 mg g^−1^, respectively. However, at higher pH levels, the negatively charged adsorbent and pollutant molecules, in the forms of TC^−^ and TC^2−^, repel each other, causing a reduction in adsorption capacity [53].

For Cr(VI), the adsorption capacity decreases as the pH increases, peaking at pH 2 with maximum capacities of 701.33, 744.15, and 752.27 mg g^−1^ for BQH, BQH-AN, and BQH-MV, respectively. Within the pH range of 1–6, hexavalent chromium primarily exists as HCrO_4_^−^ and Cr_2_O_7_^2−^, transitioning to CrO_4_^2−^ at higher pH levels. The lower free energy of HCrO_4_^−^ enhances its interaction with the active sites on the adsorbent surface, leading to higher adsorption capacity [29]. However, as the pH rises, CrO_4_^2−^ experiences electrostatic repulsion from the negatively charged adsorbent surface, while OH^−^ ions compete for the active sites, decreasing the adsorption capacity.

### 2.7. Effect of Coexisting Ions

Different ions can influence the adsorption process of biochar in real aqueous environments. Variations in ion types and strengths can modify the surface charge of the adsorbent as well as the thickness and stability of the adsorbed layer. This study examined the effect of five metal cations (Na^+^, Zn^2+^, K^+^, Ca^2+^, and Mn^2+^; added at 0.01 M) on the adsorption of pollutants by BQH, BQH-AN, and BQH-MV (Figure S10). The results show that Mn^2+^ had the most significant impact on adsorption capacity, likely because Mn, as a transition metal, exhibits multiple oxidation states that are more prone to charge transfer and can interact with the active sites on the adsorbent surface. Other metal ions did not notably affect the adsorption capacity, which may be attributed to the biochar’s stable chemical composition and the dominance of physical adsorption mechanisms, such as pore filling, over surface charge alterations [54,55]. These findings indicate that BQH, BQH-AN, and BQH-MV are relatively unaffected by ionic strength and hold great promise for practical applications in water treatment.

### 2.8. Reusability Experiments

**Figure 7:**
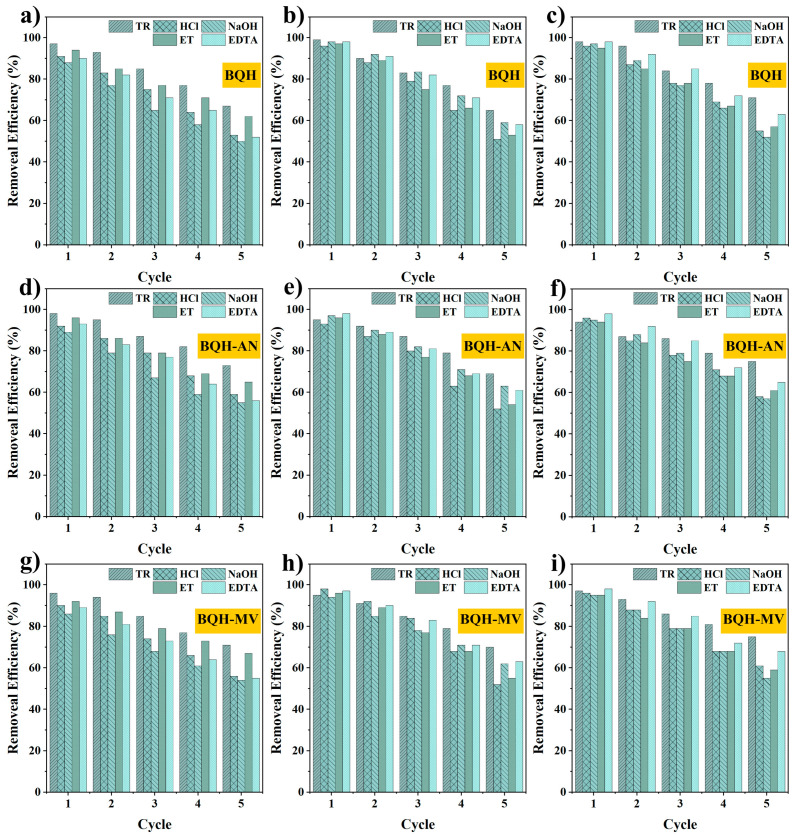
Cyclic regeneration of BQH to (**a**) RhB, (**b**) TC, and (**c**) Cr(VI). Cyclic regeneration of BQH-AN to (**d**) RhB, (**e**) TC, and (**f**) Cr(VI). Cyclic regeneration of BQH-MV to (**g**) RhB, (**h**) TC, and (**i**) Cr(VI).

The cyclic regeneration performance of adsorbents is crucial for reducing costs, minimizing environmental impacts, improving energy efficiency, and enhancing system stability and longevity. The regeneration capacity of the samples was evaluated over five cycles using four common eluents (HCl, NaOH, ethanol, and ethylenediaminetetraacetic acid) and compared to thermal regeneration methods (Figure 7). For RhB elution, ethanol (ET) showed superior performance, with BQH, BQH-AN, and BQH-MV retaining 62%, 65%, and 67% removal, respectively. This advantage may stem from ethanol’s polarity and solubility, which effectively interact with dye molecules to facilitate dissociation.

For TC, NaOH proved more effective, with the samples retaining 59%, 63%, and 64% removal, respectively. The alkaline effect of NaOH likely neutralized the acidic components of tetracycline, converting it into a negatively charged ionic form that increases water solubility and aids in elution. For Cr(VI), ethylenediaminetetraacetic acid (EDTA) was the most effective eluent, with BQH, BQH-AN, and BQH-MV retaining 57%, 61%, and 59% removal, respectively. The multiple chelation sites of EDTA allow for the formation of stable complexes with metal ions, making the elution process more resistant to dissociation or decomposition [56].

When comparing thermal regeneration to the four conventional eluent methods, thermally regenerated adsorbents exhibited higher removal rates for all three pollutants (RhB: 67%, 73%, 71%; TC: 65%, 69%, 70%; Cr(VI): 71%, 75%, 75%). This enhanced performance is likely due to the high-temperature pyrolysis process, which more effectively decomposes and removes pollutant molecules bound to the biochar’s surface. The observed decrease in adsorption capacity after five cycles may result from pore blockage by by-products generated during pollutant pyrolysis [35,36]. The superior cyclic regeneration capacity of BQH-AN and BQH-MV could be attributed to their higher thermal stability, which helps preserve pore structures. Even after five cycles, BQH-AN and BQH-MV maintained pollutant removal rates above 69%, demonstrating their strong potential for environmental water treatment applications.

### 2.9. Fixed-Bed Adsorption Column Study

**Figure 8:**
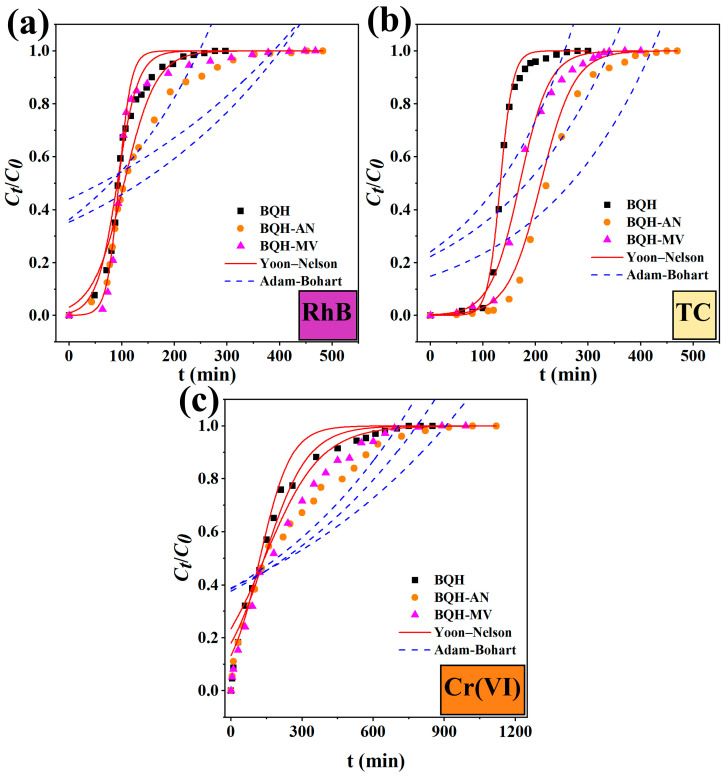
Breakthrough curves of (**a**) RhB, (**b**) TCH, and (**c**) Cr(VI) removal by BQH, BQH-AN, and BQH-MV in fixed beds at *m* = 0.1 g, *v* = 5 cm min^−1^, *C*_0_-RhB = 200 mg L^−1^, *C*_0_-TC = 200 mg L^−1^, and *C*_0_-Cr(VI) = 20 mg L^−1^.

The adsorption process in a real mobile phase was simulated through a simple fixed-bed experiment (Figure 8). The breakthrough curves for the adsorbed contaminants showed that BQH-AN and BQH-MV exhibited longer breakthrough and saturation times compared to BQH. In terms of RhB adsorption, the breakthrough time was observed to increase from 55 to 130 and 160 min, while the saturation time was found to extend from 156 to 251 and 171 min. For TC, the breakthrough time increased from 105 to 130 and 160 min, with saturation times increasing from 170 to 303 and 254 min. For Cr(VI) adsorption, the breakthrough time rose from 9 to 11 and 12 min, and the saturation time increased from 404 to 595 and 528 min.

The Adam–Bohart and Yoon–Nelson models were employed to elucidate the adsorption process occurring in the mobile phase. The higher correlation coefficients (0.93–0.99) of the Yoon–Nelson model indicate that the adsorption process is largely influenced by internal diffusion [57]. In contrast, the lower *R*^2^ values from the Adam–Bohart model suggest non-linear adsorption behavior, with external diffusion playing a less dominant role in controlling the adsorption process [58]. These experimental results suggest that the mycelial composite biochar, prepared using the bio-regulation method, demonstrates superior adsorption performance in the mobile phase and holds great promise for practical engineering applications.

### 2.10. Comparison with Other Adsorbents

In recent years, novel materials such as metal–organic frameworks (MOFs) have garnered significant attention due to their large specific surface area and pore volume. However, challenges remain regarding their preparation conditions, reaction mechanisms, and high production costs. As a result, exploring more economical and milder modification methods is still essential. The potential of the prepared biochar was evaluated by comparing the adsorption performance of BQH-AN and BQH-MV with other reported adsorbents for pollutant removal (Tables S13–S15). The data demonstrate that the mycelial composite biochar prepared via the bio-regulation method exhibits excellent adsorption properties. Furthermore, the bio-regulation approach offers milder reaction conditions and lower costs compared to chemical modification methods, which often require expensive reagents and complex synthesis steps. The outstanding performance of BQH-AN and BQH-MV in pollutant removal underscores the feasibility of using the bio-regulation method to enhance the performance of lignocellulosic biochar.

### 2.11. Probable Mechanism

**Figure 9:**
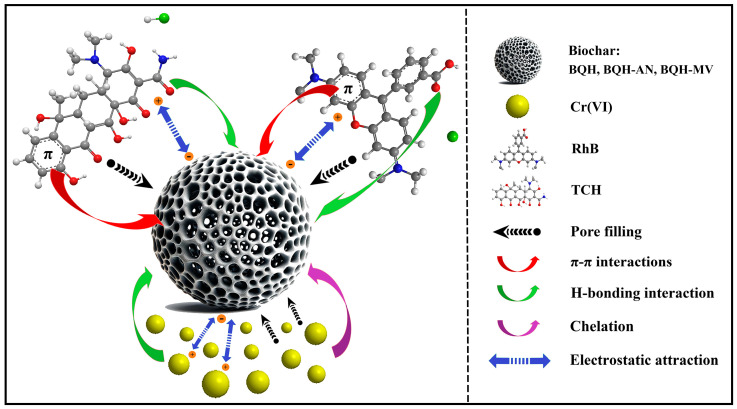
The adsorption mechanism of biochar for pollutants.

This study analyzed the potential adsorption mechanisms by comparing the N_2_ adsorption–desorption isotherms (Table S16), FT-IR (Figure S11), and XPS (Figures S12–S15) data of the biochar before and after adsorption. Pollutants gradually diffused through the pores and bound to active sites on the biochar’s inner surface, leading to a significant reduction in specific surface area and pore volume. This suggests that the pore-filling mechanism is a key factor in controlling pollutant adsorption. The π–π interaction, which occurs between π electron clouds on aromatic rings, also enhances the adsorption capacity of biochar. The biochar interacts with the π electron clouds of the aromatic pollutant molecules, facilitating π–π interactions. FT-IR spectra after adsorption revealed shifts in the absorption peak of -OH groups and the C=C/C=O stretching vibration, indicating that functional groups containing carbon and oxygen provide adsorption sites. Hydrogen bonding further plays a vital role in the adsorption process. The XPS results confirmed the successful adsorption of Cr(VI), with new Cr_2p_ absorption peaks appearing at 578.4 and 588.1 eV. Shifts in the binding energies of characteristic peaks in the O1_S_, C1_S_, and N1_S_ spectra suggest that unsaturated functional groups chemically react with Cr(VI) to form complexes, positively impacting adsorption. Moreover, the negatively charged surface of the biochar can electrostatically attract positively charged pollutants, promoting adsorption. Overall, the results indicate that the adsorption of RhB, TC, and Cr(VI) by BQH, BQH-AN, and BQH-MV is governed by multiple mechanisms (Figure 9).

## 3. Materials and Methods

### 3.1. Materials

The quinoa husk (QH) used in this study was obtained from the Black Soil Experimental Area of Jilin Agricultural University (Changchun, China, 2023). *Aspergillus niger* (AN) and *Myrothecium verrucaria* (MV) were acquired from the Key Laboratory of Straw Comprehensive Utilization and Black Soil Conservation (Changchun, China). Rhodamine B (RhB, CAS: 81-88-9), tetracycline hydrochloride (TC, CAS: 64-75-5), and Cr(VI) (potassium dichromate, CAS: 7778-50-9) were used as adsorbates and were provided by Aladdin Chemicals (Shanghai, China). Additional materials and reagents used in the experiments are listed in the Supplementary Materials S1. All reagents were of analytical grade and required no further purification.

### 3.2. Pre-Treatment of Raw Materials

Bio-regulation, a pretreatment method for biomass raw materials, involves solid-state fermentation using fungi to degrade the lignocellulosic structure. The enzymes secreted by fungi break down the lignocellulose and loosen the material’s composition, facilitating biochar preparation [59].

The QH was cleaned, dried, and sieved using an 80-mesh filter. The QH was then combined with fermentation broth (containing 0.3 g L^−1^ CaCl_2_, 2.0 g L^−1^ KH_2_PO_4_, 0.016 g L^−1^ MnSO_4_, 2.0 g L^−1^ (NH_4_)_2_SO_4_, 0.5 g L^−1^ NaCl, 0.005 g L^−1^ FeSO_4_, 0.017 g L^−1^ ZnCl_2_, and 0.3 g L^−1^ MgSO_4_) and autoclaved at 115 °C for 30 min before cooling to room temperature. Conidial suspensions (*v*/*w*) of *Aspergillus niger* (AN) and *Myrothecium verrucaria* (MV) were then introduced separately into the QH cultures. Solid-state fermentation was carried out at 29 °C, during which the fungi biodegraded the wood fibers, forming complexes with the QH through spore diffusion and growth [31]. After fermentation, the samples were freeze-dried, yielding mycelial composites named QH-AN and QH-MV.

### 3.3. Preparation of Biochar

The QH, QH-AN, and QH-MV materials were heated under nitrogen protection at a rate of 10 °C/min until they reached 600 °C, where they were held for 60 min. After cooling to room temperature, the carbonized samples were labeled CQH, CQH-AN, and CQH-MV. A total of 1.0 g of these carbonized samples was mixed with 3.0 g of solid activators (1.5 g of sodium hydroxide and 1.5 g of potassium hydroxide) in a specific ratio, thoroughly ground, and then heated to 700 °C at a rate of 10 °C min^−1^, maintaining that temperature for 60 min under nitrogen protection. After cooling, the biochar was washed with 0.1 M hydrochloric acid and deionized water, yielding the final biochar products, which were BQH, BQH-AN, and BQH-MV.

### 3.4. Adsorption Experiment

The adsorption performance of the biochar was evaluated using equilibrium adsorption experiments. In these experiments, 10 mg of biochar was evenly dispersed into 200 mL of pollutant solution within a flask. The flasks were shaken at 150 rpm in a dark temperature-controlled environment until adsorption equilibrium was reached. Afterward, the solution was extracted and diluted by centrifugation. The absorbance was measured using a UV spectrophotometer, and adsorption was calculated using the following formula [24]:

(1) $$Q_{e}=\frac{\left(C_{0}-C_{e}\right)\times V}{m}$$

In this equation, *C*_0_ represents the initial concentration of the solution (mg L^−1^), while *C_e_* denotes the equilibrium concentration (mg L^−1^). The term *m* (g) signifies the weight of the adsorbent, while *V* (L) refers to the volume of the solution.

### 3.5. Adsorption Kinetics

To assess adsorption kinetics, 10 mg of the sample was dispersed into flasks containing 200 mL of contaminant solution, with initial concentrations of 100, 200, and 300 mg L^−1^. The flasks were placed in a constant temperature shaker set to 150 rpm, and the concentration of the contaminant solution was measured at specific time intervals. The obtained adsorption data were fitted to various adsorption kinetic models, including the pseudo first-order kinetic model, pseudo second-order kinetic model, intraparticle diffusion model, and the Bangham kinetic model, to investigate potential adsorption mechanisms [50].

(2) $$Q_{t}=Q_{e}\left(1-e^{-k_{1}t}\right)$$

(3) $$Q_{t}=\frac{k_{2}Q_{e}^{2}}{\left(1+k_{2}Q_{e}t\right)}t$$

(4) $$Q_{t}=k_{3}t^{0.5}+C$$

(5) $$Q_{t}=Q_{e}\left(1-e^{-k_{4}t^{n}}\right)$$

where *n* represents the boundary layer thickness and *Q_t_* is the adsorption capacity of the sample at different time points. *k*_1_, *k*_2_, *k*_3_, and *k*_4_ represent the rate constants of the above kinetic model, respectively.

### 3.6. Adsorption Isotherm

Adsorption isotherm experiments were conducted at various temperatures (293, 303, and 313 K), with initial pollutant solution concentrations of 100, 200, and 300 mg L^−1^. In each experiment, 10 mg of the sample was dispersed into flasks containing 200 mL of contaminant solution. The flasks were placed on a constant temperature shaker at 150 rpm and kept in the dark until adsorption equilibrium was reached. The adsorption data were then fitted using the Langmuir, Freundlich, Temkin, and Liu isotherm models. These models helped analyze the equilibrium relationship between the biochar and the pollutants during the adsorption process [52,60].

(6) $$Q_{e}=\frac{Q_{m}K_{L}C_{e}}{1+K_{L}C_{e}}$$

(7) Qe=KFCe1nF

(8) $$Q_{e}=\frac{RT}{b\left(lnAC_{e}\right)}$$

(9) $$Q_{e}=\frac{Q_{m}{\left(K_{g}C_{e}\right)}^{n_{L}}}{1+{\left(K_{g}C_{e}\right)}^{n_{L}}}$$

In this context, *Q_m_* represents the theoretical maximum value of adsorption, while *K_L_*, *K_F_*, and *K_g_* correspond to the constant maturation values of the three models under consideration. Furthermore, the exponents *n_F_* and *n_L_* correspond to the Freundlich and Langmuir models, respectively. *R* represents the gas constant, and *b* and *A* represent the heat of adsorption and binding energy in the model.

### 3.7. Adsorption Thermodynamics

In this experiment, 10 mg of the sample was dispersed into flasks containing 200 mL of contaminant solution with varying initial concentrations. The adsorption process was conducted in a constant temperature shaker at 150 rpm under different temperatures (293, 303, and 313 K). Once equilibrium was reached, the solution concentration was determined and the thermodynamic parameters were calculated [55].

(10) $$ln(\frac{Q_{e}}{C_{e}})=\frac{\Delta S}{R}-\frac{\Delta H}{RT}$$

(11) ΔG=ΔH−TΔS

where *T* (K) is the absolute temperature and *R* is 8.314 J mol^−1^ K^−1^.

### 3.8. Effect of pH and Ionic Strength

Equilibrium adsorption experiments were conducted by adjusting the pH of the contaminant solutions using 0.1 M HCl and 0.1 M NaOH. For each experiment, 10 mg of biochar was dispersed into a flask containing 200 mL of contaminant solution with varying pH values. The flasks were shaken on a thermostatic shaker at 150 rpm under dark conditions until equilibrium was reached. The concentration of contaminants was then measured using a UV spectrophotometer, and adsorption values were calculated.

The effect of different cations on the adsorption of pollutants by biochar was evaluated by preparing pollutant solutions containing different cations, such as NaCl, ZnSO_4_, K_2_SO_4_, CaCl_2_, and MgSO_4_ [61]. To this end, 10 mg of biochar was dispersed into a flask containing 200 mL of contaminant solution, and the flasks were shaken in the dark on a thermostatic shaker (150 rpm) until equilibrium was reached. The concentration of the pollutant solution was determined using a UV spectrophotometer.

### 3.9. Reusability Assessment

The cyclic regeneration performance of biochar is a key factor in determining its practical applicability. To assess this, the biochar was regenerated using EDTA, anhydrous ethanol, hydrochloric acid, and sodium hydroxide solutions as eluents. Additionally, thermal regeneration was tested by heating the adsorbed biochar to 500 °C under nitrogen protection for 60 min. After cooling to room temperature, the regenerated biochar was used in subsequent adsorption cycles [62].

### 3.10. Fixed-Bed Column Experiment

A fixed-bed column experiment was conducted to assess the ability of biochar to adsorb pollutants in a dynamic mobile phase system. For this experiment, 0.1 g of biochar was used, with RhB and TC solutions at concentrations of 200 mg L^−1^ and potassium dichromate at 20 mg L^−1^. Breakthrough and saturation points were defined at a ratio of C_t_/C_0_ of 10% and 90%, respectively. The adsorption data were subjected to fitting using the Yoon–Nelson and Adam–Bohart models [63].

(12) $$\frac{C_{t}}{C_{0}}=\frac{1}{1+exp^{k\left(t-A\right)}}$$

(13) $$\frac{C_{t}}{C_{0}}=\frac{1}{1+exp[k_{AB}C_{0}(\frac{ZN_{0}}{UC_{0}}-t)]}$$

where k and k_AB_ are the rate constants of the model and Z and U represent the bed height and linear velocity. A is the time required for the permeate concentration to reach 50%. N_0_ (mg/L) is the adsorption capacity of adsorbent per unit volume of the bed.

### 3.11. Statistical Error Analysis

To evaluate the accuracy of the fitted models, the standard deviation of residuals (SDE), sum of squared errors (SSE), and Bayesian information criterion (BIC) were employed. Lower values of the SSE, SDE, and BIC, along with higher R^2^ values, indicate a better fit of the kinetic and isotherm models to the experimental results. The SSE, SDE, and BIC were calculated using the following equations [27]:

(14) $$SSE=\sum_{i=1}^{n}{\left(Q_{e,i}-Q_{e,i,cat.}\right)}^{2}$$

(15) $$SD=\sqrt{(\frac{1}{n-p})\times \sum_{i=1}^{n}{\left(Q_{e,i}-Q_{e,i,cat.}\right)}^{2}}$$

(16) $$BIC=n\times ln(\frac{SSE}{n})+p\times ln\left(n\right)$$

where *Q_e,i_* (mg g^−1^), *Q_e,i,cat._* (mg g^−1^), *n*, and *p* represent the experimental adsorption capacity, the theoretical adsorption capacity, the number of experiments, and the number of parameters used in the model, respectively.

### 3.12. Characterization Tests

Detailed information regarding the sample characterization tests can be found in the Supplementary Materials S1.2.

## 4. Conclusions

In this study, mycelial composite biochar materials (BQH-AN and BQH-MV) with stable physicochemical properties and good adsorption performance were prepared from quinoa hulls through bio-modification. Specifically, quinoa hulls were modified via microbial solid fermentation, which disrupted the compact wood fiber structure of the quinoa hulls and introduced a large number of active functional groups and unsaturated functional valences. Characterization results indicated that the specific surface area and total pore volume of the biochar BQH-AN and BQH-MV, prepared through microbial modification, increased by 21.3–34.3% and 30.9–35.9%, respectively, compared to the original quinoa husk biochar. Additionally, the biochar performed well in adsorption experiments with RhB, TC, and Cr(VI), showing that the maximum adsorption capacity of the mycelial composite biochar was significantly higher than that of the pristine biochar (BQH-RhB: 1134.78 mg g^−1^, BQH-TC: 1280.88 mg g^−1^, BQH-Cr(VI): 701.33 mg g^−1^; BQH-AN-RhB: 1450.79 mg g^−1^, BQH-AN-TC: 1608.43 mg g^−1^, BQH-AN-Cr(VI): 744.15 mg g^−1^; BQH-MV-RhB: 1329.85 mg g^−1^, BQH-MV-TC: 1526.46 mg g^−1^, BQH-MV-Cr(VI): 752.27 mg g^−1^). The mechanisms of pollutant adsorption on biochar were analyzed through adsorption kinetics, isotherms, and other experiments, revealing that the adsorption process was controlled by pore filling, π–π interactions, hydrogen bonding, complexation, and electrostatic attraction, with pore filling likely playing a major role. The excellent performance of BQH-AN and BQH-MV demonstrates the feasibility of using the bio-regulation method to enhance the properties of lignocellulosic biochar. This method is relatively mild and simple, suggesting potential applications in areas beyond adsorption, such as supercapacitors and electrocatalysis. Currently, the principles of bio-modification are not yet clear, and the process still needs to be further optimized, as the bio-modification process often involves long processing times and high environmental requirements. However, this green, gentle, and simple modification method has great potential in other fields, such as supercapacitors, electrocatalysis, and energy storage, which motivates us to conduct more in-depth research to advance its implementation in large-scale process production.

## Data Availability

The original contributions presented in the study are included in the article/Supplementary Materials, further inquiries can be directed to the corresponding authors.

## Supplementary Materials

The following supporting information can be downloaded at: https://www.mdpi.com/article/10.3390/ijms252111732/s1. References [64,65,66,67,68,69,70,71,72,73,74,75,76,77,78,79,80,81,82,83,84,85,86,87,88,89,90,91,92,93,94,95,96,97,98,99,100,101,102,103,104,105,106,107,108] are cited in the Supplementary Materials.

## References

[1] Garai S., Bhattacharjee C., Sarkar S., Moulick D., Dey S., Jana S., Dhar A., Roy A., Mondal K., Mondal M. (2024). Microplastics in the soil–water–food nexus: Inclusive insight into global research findings. Sci. Total Environ..

[2] Liu X., Wang Y., Liu H., Zhang Y., Zhou Q., Wen X., Guo W., Zhang Z. (2024). A systematic review on aquaculture wastewater: Pollutants, impacts, and treatment technology. Environ. Res..

[3] Vo T.P., Danaee S., Chaiwong C., Pham B.T., Poddar N., Kim M., Kuzhiumparambil U., Songsomboon C., Pernice M., Ngo H.H. (2024). Microalgae-bacteria consortia for organic pollutants remediation from wastewater: A critical review. J. Environ. Chem. Eng..

[4] Xuan X., Chen H., Li H., Wei C., Jiang Y., Zeng S., Zhang C., Zhang W., Jin Y. (2024). Research on emerging pollutants in wastewater: Bibliometric analysis. Desalin. Water Treat..

[5] Ding S., Li X., Qiao X., Liu Y., Wang H., Ma C. (2024). Identification and screening of priority pollutants in printing and dyeing industry wastewater and the importance of these pollutants in environmental management in China. Environ. Pollut..

[6] Pour S.E., Mamaghani A.H., Hashisho Z. (2025). Modeling of adsorption process on monolith adsorbents: A mini-review. Sep. Purif. Technol..

[7] Wang Q., Tang S., Zhang Y., Lai C.J. (2024). Recent development of natural polysaccharide-modified biochar on adsorption of pollutants from wastewater: Preparation, characterization, mechanisms and applications. Sep. Purif. Technol..

[8] Chu Y., Wang Z., Wang W., Zeng Y., He S., Yan C., Qin F., Wu M., Zeng G., Zhou C. (2025). A review on the algae-derived biochar catalysts: Advanced oxidation processes and machine learning tools. Sep. Purif. Technol..

[9] Zhang J., Gu J., Shan R., Yuan H., Chen Y. (2025). Advances in thermochemical valorization of biomass towards carbon neutrality. Resour. Conserv. Recycl..

[10] Saravanakumar A., Vijayakumar P., Hoang A.T., Kwon E.E., Chen W.-H. (2023). Thermochemical conversion of large-size woody biomass for carbon neutrality: Principles, applications, and issues. Bioresour. Technol..

[11] Yang E., Chon K., Kim K.Y., Le G.T.H., Nguyen H.Y., Le T.T.Q., Nguyen H.T.T., Jae M.R., Ahmad I., Oh S.E. (2023). Pretreatments of lignocellulosic and algal biomasses for sustainable biohydrogen production: Recent progress, carbon neutrality, and circular economy. Bioresour. Technol..

[12] Ruello J.L.A., Mengesha D.N., Choi Y., Baye A.F., Kim H. (2024). Laser-cum-KOH activation allows interfacial engineering of cardboard-derived carbon, tunable ionic states, and universal dye adsorption. Chemosphere.

[13] Romero-Hernandez J.J., Paredes-Laverde M., Silva-Agredo J., Mercado D.F., Ávila-Torres Y., Torres-Palma R.A. (2024). Pharmaceutical adsorption on NaOH-treated rice husk-based activated carbons: Kinetics, thermodynamics, and mechanisms. J. Clean. Prod..

[14] Zhao T., Ali A., Su J., Liu S., Yan H., Xu L. (2024). Removal of sulfamethoxazole from water by biosurfactant-modified sludge biochar: Properties and mechanism. J. Environ. Chem. Eng..

[15] Wang T., Zhang D., Fang K., Zhu W., Peng Q., Xie Z. (2021). Enhanced nitrate removal by physical activation and Mg/Al layered double hydroxide modified biochar derived from wood waste: Adsorption characteristics and mechanisms. J. Environ. Chem. Eng..

[16] Lamberti E., Viscusi G., Kiani A., Boumezough Y., Acocella M.R., Gorrasi G. (2024). Efficiency of dye adsorption of modified biochar: A comparison between chemical modification and ball milling assisted treatment. Biomass Bioenergy.

[17] Miao Q., Li G. (2021). Potassium phosphate/magnesium oxide modified biochars: Interfacial chemical behaviours and Pb binding performance. Sci. Total Environ..

[18] Ghorbani M., Konvalina P., Neugschwandtner R.W., Soja G., Bárta J., Chen W.-H., Amirahmadi E. (2024). How do different feedstocks and pyrolysis conditions effectively change biochar modification scenarios? A critical analysis of engineered biochars under H_2_O_2_ oxidation. Energy Convers. Manag..

[19] Yu Y., Zhang H., Zhang Y., Zhang B., Jin Y., Chen S., Liang D., Tang S., Li J., Chen G. (2024). Fungus-mediated preparation of porous carbon based on wheat straw: Efficient removal of chromium and enhanced electrochemical properties. Ind. Crop. Prod..

[20] Xia Y., Zhang B., Guo Z., Tang S., Su Y., Yu X., Chen S., Chen G. (2022). Fungal mycelium modified hierarchical porous carbon with enhanced performance and its application for removal of organic pollutants. J. Environ. Chem. Eng..

[21] Nie Y., Zhao C., Zhou Z., Kong Y., Ma J. (2023). Hydrochloric acid-modified fungi-microalgae biochar for adsorption of tetracycline hydrochloride: Performance and mechanism. Bioresour. Technol..

[22] Huang Y., Liu T., Liu J., Xiao X., Wan Y., An H., Luo X., Luo S. (2024). Exceptional anti-toxic growth of water spinach in arsenic and cadmium co-contaminated soil remediated using biochar loaded with *Bacillus aryabhattai*. J. Hazard. Mater..

[23] Li B., Liu Y., Tong W.K., Tang H., Wang W., Gao M., Dai C., Liu N., Hu J., Li J. (2024). Effects of cellulase treatment on properties of lignocellulose-based biochar. Bioresour. Technol..

[24] Silva N.E.P., Bezerra L.C.A., Araújo R.F., Moura T.A., Vieira L.H.S., Alves S.B.S., Fregolente L.G., Ferreira O.P., Avelino F. (2024). Coconut shell-based biochars produced by an innovative thermochemical process for obtaining improved lignocellulose-based adsorbents. Int. J. Biol. Macromol..

[25] Nouri N., Sadeghi L., Marefat A. (2024). Production of alkaline protease by *Aspergillus niger* in a new combinational paper waste culture medium. J. Biosci. Bioeng..

[26] Liu P., Wang Y., Kang H., Wang Y., Yu H., Peng H., He B., Xu C., Jia K., Liu S. (2024). Upgraded cellulose and xylan digestions for synergistic enhancements of biomass enzymatic saccharification and bioethanol conversion using engineered *Trichoderma reesei* strains overproducing mushroom LeGH7 enzyme. Int. J. Biol. Macromol..

[27] Wang Q., Niu L., Jiao J., Guo N., Zang Y., Gai Q., Fu Y. (2017). Degradation of lignin in birch sawdust treated by a novel *Myrothecium verrucaria* coupled with ultrasound assistance. Bioresour. Technol..

[28] Rouzitalab Z., Maklavany D.M., Jafarinejad S., Rashidi A. (2020). Lignocellulose-based adsorbents: A spotlight review of the effective parameters on carbon dioxide capture process. Chemosphere.

[29] Zhang J., Wang S., Cheng X., Chen C., Zhang L., Wang Z. (2025). Design strategies and advantages of metal-organic frameworks@lignocellulose-based composite aerogel for CO_2_ capture: A review. Sep. Purif. Technol..

[30] Xia Y., Jin Y., Qi J., Chen H., Chen G., Tang S. (2021). Preparation of biomass carbon material based on *Fomes fomentarius* via alkali activation and its application for the removal of brilliant green in wastewater. Environ. Technol. Innov..

[31] Jin Y., Zhang B., Chen G., Chen H., Tang S. (2022). Combining biological and chemical methods to disassemble of cellulose from corn straw for the preparation of porous carbons with enhanced adsorption performance. Int. J. Biol. Macromol..

[32] Cao X., Zuo S., Cai R., Yang F., Jiang X., Xu C. (2023). Expansion combined with *Irpex lacteus* fungal treatment for enhancing buckwheat straw degradation. Biochem. Eng. J..

[33] Averheim A., Reis G.S., Grimm A., Bergna D., Heponiemi A., Lassi U., Thyrel M. (2024). Enhanced biobased carbon materials made from softwood bark via a steam explosion preprocessing step for reactive orange 16 dye adsorption. Bioresour. Technol..

[34] Li Z., Tong W., Li C., Dong Z., Han S., Li K., Wang J., Qu J., Zhang Y. (2025). Advances on nitrogen-doped biochar for adsorption and degradation of organic pollutants from aquatic environment: Mechanisms and applications. Sep. Purif. Technol..

[35] Yu D., He Y., Zeng S., Tian H., Ji Z. (2024). A novel magnetic S/N co-doped tea residue biochar applied to tetracycline adsorption in water environment. Colloids Surf. A.

[36] Chen S., Xia Y., Zhang B., Chen H., Chen G., Tang S. (2021). Disassembly of lignocellulose into cellulose, hemicellulose, and lignin for preparation of porous carbon materials with enhanced performances. J. Hazard. Mater..

[37] Su Y., Xie K., Xiao J., Chen S. (2022). Influence of microbial treatment on the preparation of porous biochar with stepped-up performance and its application in organic pollutants control. Int. J. Mol. Sci..

[38] Srivastava N., Singh R., Verma B., Rai A.K., Tripathi S.C., Bantun F., Faidah H., Singh R.P., Jalal N.A., Abdel-Razik N.E. (2023). Microbial cellulase production and stability investigations via graphene like carbon nanostructure derived from paddy straw. Int. J. Biol. Macromol..

[39] Wang Z., Dong Y., Zhou T., Wu Y., Tan Z. (2024). Exploring the mechanism of removing Cd from polluted soil using electrochemical leaching with straw carbon film electrode. J. Environ. Chem. Eng..

[40] Yang X., Lotfy V.F., Basta A.H., Liu H., Fu S. (2024). Carbon quantum dots derived from rice straw doped with N and S and its nanocomposites with hydroxypropyl cellulose nanocomposite. Int. J. Biol. Macromol..

[41] Ding S., Zeng X., Wang B., Yan Y., Ren B., Li Y., Yang X. (2024). Synthesis of corn straw based carbon doped Nb_2_O_5_ as photocatalysts for Rhodamine B degradation under visible light illumination. Inorg. Chem. Commun..

[42] Wang X., Ma T., Liu H., Liu Y., Yu B., Chen Y., Wu L., Zhai M., Zhou H. (2025). In-depth understanding of high temperature and low residence time on the corn straw rapid pyrolysis char structure evolution. Fuel.

[43] Lv C., Liu P., Cheng S. (2024). Preparation of biochar from pyrolysis of soybean straw at different pyrolysis temperature for cadmium removal from wastewater and pyrolysis gas investigation. Arab. J. Chem..

[44] Chai B., Xiao T., Xiao E., Du S., Yang S., Yin H., Dang Z., Pan K. (2024). Enhancing microplastics removal from soils using wheat straw and cow dung-derived biochars. J. Clean. Prod..

[45] Ji S., Cao G., Lv H., Gao P., Wang C. (2024). Monolithic TS-1 prepared with nitrogen-doped carbon nanotubes loaded on nickel foam as carriers and the structure-activity relationships in propylene epoxidation. Mol. Catal..

[46] Zhu K., Shen Y., Hou J., Gao J., He D., Huang J., He H., Lei L., Chen W. (2021). One-step synthesis of nitrogen and sulfur co-doped mesoporous graphite-like carbon nanosheets as a bifunctional material for tetracycline removal via adsorption and catalytic degradation processes: Performance and mechanism. Chem. Eng. J..

[47] Liu Z., Yang F., Zhai T., Yu J., Wang C., Liu Z., Liu Z., Gao Y., Yang M. (2024). Removal of PFOA from water by activated carbon adsorption: Influence of pore structure. J. Environ. Chem. Eng..

[48] Xiong X., Li Y., Zhang C. (2024). Enhanced phosphorus removal from anoxic water using oxygen-carrying iron-rich biochar: Combined roles of adsorption and keystone taxa. Water Res..

[49] Saigl Z.M., Aljuaid O.A. Adsorption of Rhodamine B dye onto iodo-polyurethane foam: Kinetics and thermodynamic study. 2023, 315, 227–240.

[50] Liang J., Zhang W., Yao X., Chen M., Chen X., Kong L., Diao Z. (2023). New insights into co-adsorption of Cr^6+^ and chlortetracycline by a new fruit peel based biochar composite from water: Behavior and mechanism. Colloids Surf. A.

[51] Melliti A., Yılmaz M., Sillanpaa M., Hamrouni B., Vurm R. (2023). Low-cost date palm fiber activated carbon for effective and fast heavy metal adsorption from water: Characterization, equilibrium, and kinetics studies. Colloids Surf. A.

[52] Zhang J., Hu X., Yan X., Feng R., Zhou M., Xue J. (2019). Enhanced adsorption of Rhodamine B by magnetic nitrogen-doped porous carbon prepared from bimetallic ZIFs. Colloids Surf. A.

[53] Xian B., Tang W., Xiang D., Rao C., Liu X., Fang F., Chu F., Fang T. (2024). Preparation of organic potassium salts modified microalgae biochar and its high-efficient removal of tetracycline hydrochloride from water: Activation mechanism and adsorption mechanism. J. Water Process. Eng..

[54] Nguyen V.K., Nguyen T.H.H., Pham T.D., Truong T.T. (2024). Adsorption characteristics and mechanisms of individual and a quinary mixture of heavy metal ions on novel CoFe_2_O_4_-BiFeO_3_ nanosorbents in water. Inorg. Chem. Commun..

[55] Li Z., Qiu J., Xu X., Wan R., Yao M., Wang H., Zhou Z., Xu J. (2025). Solar driven kaolin-based hydrogels for efficient interfacial evaporation and heavy metal ion adsorption from wastewater. Sep. Purif. Technol..

[56] Al-Amrani W.A., Onaizi S.A. (2024). Adsorptive removal of heavy metals from wastewater using emerging nanostructured materials: A state-of-the-art review. Sep. Purif. Technol..

[57] Chen K.-H., Lai Y.-R., Hanh N.T.D. (2024). Breakthrough curve modeling for lysozyme by ion-exchange nanofiber membrane: Linear and nonlinear analysis. J. Taiwan Inst. Chem. Eng..

[58] Zheng C., Yang X., Kang K., Xie Y., Tang M., Song H., Liang Y., Hu J., Bai S. (2025). Comparison and development of the descriptive model with polynomial structures to fit multi-component dynamic breakthrough curves with roll-up, stepwise, and saddle-shaped structure. Sep. Purif. Technol..

[59] Liang H., Ding W., Zhang H., Peng P., Peng F., Geng Z., She D., Li Y. (2022). A novel lignin-based hierarchical porous carbon for efficient and selective removal of Cr(VI) from wastewater. Int. J. Biol. Macromol..

[60] Shen Y., Zhang N. (2022). A facile synthesis of nitrogen-doped porous carbons from lignocellulose and protein wastes for VOCs sorption. Environ. Res..

[61] Reguyal F., Sarmah A.K. (2018). Adsorption of sulfamethoxazole by magnetic biochar: Effects of pH, ionic strength, natural organic matter and 17α-ethinylestradiol. Sci. Total Environ..

[62] Chen K., Song C., Huang Z., Rao L., Jin X., Liu G., He F., Huang Q. (2023). Thermally regenerable FeS/N-doped biochar catalyzed peroxydisulfate oxidative destruction of aqueous triclosan. Chem. Eng. J..

[63] Deng Y., Chen J., She A., Ni F., Chen W., Ao T., Zhang Y. (2024). A novel Fe-loaded porous hydrothermal biochar for removing tetracycline from wastewater: Performance, mechanism, and fixed-bed column. J. Environ. Chem. Eng..

[64] Li X., Shi J., Luo X. (2022). Enhanced adsorption of rhodamine B from water by Fe-N co-modified biochar: Preparation, performance, mechanism and reusability. Bioresour. Technol..

[65] Xiao W., Garba Z.N., Sun S., Lawan I., Wang L., Lin M., Yuan Z. (2020). Preparation and evaluation of an effective activated carbon from white sugar for the adsorption of rhodamine B dye. J. Clean. Prod..

[66] Maneerung T., Liew J., Dai Y., Kawi S., Chong C., Wang C. (2016). Activated carbon derived from carbon residue from biomass gasification and its application for dye adsorption: Kinetics, isotherms and thermodynamic studies. Bioresour. Technol..

[67] Wang S., Yang B., Liu Y. (2017). Synthesis of a hierarchical SnS2 nanostructure for efficient adsorption of Rhodamine B dye. J. Colloid Interf. Sci..

[68] Liu K., Li H., Wang Y., Gou X., Duan Y. (2015). Adsorption and removal of rhodamine B from aqueous solution by tannic acid functionalized graphene. Colloids Surf. A.

[69] Wang Y.S., Huo T.R., Wang Y., Bai J.W., Huang P.P., Li C., Deng S.Y., Mei H., Qian J., Zhang X.C. (2024). Constructing mesoporous biochar derived from waste carton: Improving multi-site adsorption of dye wastewater and investigating mechanism. Environ. Res..

[70] Anandkumar J., Mandal B. (2011). Adsorption of chromium(VI) and Rhodamine B by surface modified tannery waste: Kinetic, mechanistic and thermodynamic studies. J. Hazard. Mater..

[71] Hayeeye F., Sattar M., Chinpa W., Sirichote O. (2017). Kinetics and thermodynamics of Rhodamine B adsorption by gelatin/activated carbon composite beads. Colloids Surf. A.

[72] Gad H.M.H., El-Sayed A.A. (2009). Activated carbon from agricultural by-products for the removal of Rhodamine-B from aqueous solution. J. Hazard. Mater..

[73] Yu J., Li B., Sun X., Yuan J., Chi R. (2009). Polymer modified biomass of baker’s yeast for enhancement adsorption of methylene blue, rhodamine B and basic magenta. J. Hazard. Mater..

[74] Indujalekshmi J., Arsha M.S., Biju V. (2018). KOH-mediated structural modification of activated charcoal by heat treatment for the efficient adsorption of organic dyes. J. Environ. Manag..

[75] Wang X., Chen S., Sun J., Zhang D., Yan Z., Xu X. (2021). Synthesis of large pore sized mesoporous carbon using alumina-templated strategy for high-performance RhB removal. Microporous Mesoporous Mater..

[76] Huang Y., Zheng X., Feng S., Guo Z., Liang S. (2016). Enhancement of rhodamine B removal by modifying activated carbon developed from *Lythrum salicaria* L. with pyruvic acid. Colloids Surf. A.

[77] Zhang T., Huang J. (2017). N-vinylimidazole modified hyper-cross-linked resins and their adsorption toward Rhodamine B: Effect of the cross-linking degree. J. Taiwan Inst. Chem. E..

[78] Somsiripan T., Sangwichien C. (2023). Enhancement of adsorption capacity of Methylene blue, Malachite green, and Rhodamine B onto KOH activated carbon derived from oil palm empty fruit bunches. Arab. J. Chem..

[79] Gong J., Liu R., Sun Y., Xu J., Liang M., Sun Y., Long L. (2024). Preparation of high-performance nitrogen doped porous carbon from cork biomass by K_2_CO_3_ activation for adsorption of rhodamine B. Ind. Crop. Prod..

[80] Wang Q., He D., Li C., Sun Z., Mu J. (2023). Honeycomb-like cork activated carbon modified with carbon dots for high-efficient adsorption of Pb(II) and rhodamine B. Ind. Crop. Prod..

[81] Gao X., Zheng M., Zhao X., Song S., Gao Z. (2021). Ultra-High-Capacity Adsorption of Rhodamine B in a Carboxyl-Functionalized Metal-Organic Framework via Surface Adsorption. J. Chem. Eng. Data.

[82] Chen Y., Wang F., Duan L., Yang H., Gao J. (2016). Tetracycline adsorption onto rice husk ash, an agricultural waste: Its kinetic and thermodynamic studies. J. Mol. Liq..

[83] Premarathna K.S.D., Rajapaksha A.U., Adassoriya N., Sarkar B., Sirimuthu N.M.S., Cooray A., Ok Y.S., Vithanage M. (2019). Clay-biochar composites for sorptive removal of tetracycline antibiotic in aqueous media. J. Environ. Manag..

[84] Yu H., Gu L., Chen L., Wen H., Zhang D., Tao H. (2020). Activation of grapefruit derived biochar by its peel extracts and its performance for tetracycline removal. Bioresour. Technol..

[85] Huang H., Niu Z., Shi R., Tang J., Lv L., Wang J., Fan Y. (2020). Thermal oxidation activation of hydrochar for tetracycline adsorption: The role of oxygen concentration and temperature. Bioresour. Technol..

[86] Zhang D., He Q., Hu X., Zhang K., Chen C., Xue Y. (2021). Enhanced adsorption for the removal of tetracycline hydrochloride (TC) using ball-milled biochar derived from crayfish shell. Colloids Surf. A.

[87] Xiang W., Wan Y., Zhang X., Tan Z., Xia T., Zheng Y., Gao B. (2020). Adsorption of tetracycline hydrochloride onto ball-milled biochar: Governing factors and mechanisms. Chemosphere.

[88] Oladipo A.A., Ifebajo A.O. (2018). Highly efficient magnetic chicken bone biochar for removal of tetracycline and fluorescent dye from wastewater: Two-stage adsorber analysis. J. Environ. Manag..

[89] Zhou Y., He Y., He Y., Liu X., Xu B., Yu J., Dai C., Huang A., Pang Y., Luo L. (2019). Analyses of tetracycline adsorption on alkali-acid modified magnetic biochar: Site energy distribution consideration. Sci. Total Environ..

[90] Miao J.H., Wang F.H., Chen Y.J., Zhu Y.Z., Zhou Y., Zhang S.T. (2019). The adsorption performance of tetracyclines on magnetic graphene oxide: A novel antibiotics absorbent. Appl. Surf. Sci..

[91] Yang Z., Zhao Z., Yang X., Ren Z. (2021). Xanthate modified magnetic activated carbon for efficient removal of cationic dyes and tetracycline hydrochloride from aqueous solutions. Colloids Surf. A.

[92] Gao Y., Li Y., Zhang L., Huang H., Hu J., Shah S.M., Su X. (2012). Adsorption and removal of tetracycline antibiotics from aqueous solution by graphene oxide. J. Colloid Interface Sci..

[93] Martins A., Pezoti O., Cazetta A., Bedin K., Yamazaki D., Bandoch G., Asefa T., Visentainer J., Almeida V. (2015). Removal of tetracycline by NaOH-activated carbon producedfrom macadamia nut shells: Kinetic and equilibrium studies. Chem. Eng. J..

[94] Yang G., Gao Q., Yang S., Yin S., Cai X., Yu X., Zhang S., Fang Y. (2020). Strong adsorption of tetracycline hydrochloride on magnetic carbon-coated cobalt oxide nanoparticles. Chemosphere.

[95] Chen Y., Liu J., Zeng Q., Liang Z., Ye X., Lv Y., Liu M. (2021). Preparation of Eucommia ulmoides lignin-based high-performance biochar containing sulfonic group: Synergistic pyrolysis mechanism and tetracycline hydrochloride adsorption. Bioresour. Technol..

[96] Masoumi S., Tabrizi F.F., Sardarian A.R. (2020). Efficient tetracycline hydrochloride removal by encapsulated phosphotungstic acid (PTA) in MIL-53 (Fe): Optimizing the content of PTA and recycling study. J. Environ. Chem. Eng..

[97] Perera H.M., Rajapaksha A.U., Liyanage S., Ekanayake A., Selvasembian R., Daverey A., Vithanage M. (2023). Enhanced adsorptive removal of hexavalent chromium in aqueous media using chitosan-modified biochar: Synthesis, sorption mechanism, and reusability. Environ. Res..

[98] Xiao F., Cheng J., Cao W., Yang C., Chen J., Luo Z. (2019). Removal of heavy metals from aqueous solution using chitosan-combined magnetic biochars. J. Colloid Interface Sci..

[99] Altun T., Ecevit H., Kar Y., Çiftçi B. (2021). Adsorption of Cr(VI) onto cross-linked chitosan-almond shell biochars: Equilibrium, kinetic, and thermodynamic studies. J. Anal. Sci. Technol..

[100] Luo L., Cheng S., Yue L., You Z., Cai J. (2022). N-doped biochar from chitosan gel-like solution: Effect of hydrothermal temperature and superior aqueous Cr (VI) removal performance. Colloids Surf. A Physicochem. Eng. Asp..

[101] Yang Y., Zhang Y., Wang G., Yang Z., Xian J., Yang Y., Li T., Pu Y., Jia Y., Li Y. (2021). Adsorption and reduction of Cr(VI) by a novel nanoscale FeS/chitosan/biochar composite from aqueous solution. J. Environ. Chem. Eng..

[102] Wang B., Zeng Y., Xiong M., Qiu R. (2023). Adsorption performance and mechanism of mesoporous carbon-doped Al_2_O_3_ adsorbent derived from NH_2_-MIL-53 (Al) for removing Cr(VI) and methyl orange from aqueous solution. J. Environ. Chem. Eng..

[103] Wang H., Wang W., Zhou S., Gao X. (2023). Adsorption mechanism of Cr(VI) on woody-activated carbons. Heliyon.

[104] Kharrazi S.M., Soleimani M., Jokar M., Richards T., Pettersson A., Mirghaffari N. (2021). Pretreatment of lignocellulosic waste as a precursor for synthesis of high porous activated carbon and its application for Pb (II) and Cr (VI) adsorption from aqueous solutions. Int. J. Biol. Macromol..

[105] Xu H., Liu Y., Liang H., Gao C., Qin J., You L., Wang R., Li J., Yang S. (2021). Adsorption of Cr(VI) from aqueous solutions using novel activated carbon spheres derived from glucose and sodium dodecylbenzene sulfonate. Sci. Total Environ..

[106] Chen X., Song Z., Yuan B., Li X., Li S., Nguyen T.T., Guo M., Guo Z. (2022). Fluorescent carbon dots crosslinked cellulose Nanofibril/Chitosan interpenetrating hydrogel system for sensitive detection and efficient adsorption of Cu (II) and Cr (VI). Chem. Eng. J..

[107] Liang H., Ma K., Zhao X., Geng Z., She D., Hu H. (2023). Enhancement of Cr(VI) adsorption on lignin-based carbon materials by a two-step hydrothermal strategy: Performance and mechanism. Int. J. Biol. Macromol..

[108] Yang X., Wang B., Zhang P., Song X. (2024). Adsorption and reduction of Cr(VI) by N, S co-doped porous carbon from sewage sludge and low-rank coal: Combining experiments and theoretical calculations. Sci. Total Environ..

