# Supplementary material for: Biochar Composite with Enhanced Performance Prepared Through Microbial Modification for Water Pollutant Removal
Source: Int J Mol Sci. 2024 Oct 31;25(21):11732. doi: 10.3390/ijms252111732 (PMC11546741; doi:10.3390/ijms252111732)
Supplement: Supplementary file 1 [file ijms-25-11732-s001.zip › ijms-3261300-supplementary.pdf]

Biochar Composite with Enhanced Performance Prepared Through Microbial Modification for Water Pollutant Removal

Bolun Zhang[a,b], Ruqi Li[a,b], Yangyang Zheng[a,b], Siji Chen[a,b,*], Yingjie Su[a,b,*], Wei Zhou[a,b], Qi Sui[a,b] and Dadong Liang[a,b]

[a] Jilin Agricultural University, College of Life Sciences, Changchun 130118, China;

[b] Key Laboratory of Straw Comprehensive Utilization and Black Soil Conservation, Ministry of Education, Jilin Agricultural University, Changchun 130118, China.

Author E-mail: Author E-mail: aa921526347@163.com, 14752267617@163.com, z08130214@163.com, zhouwei6423@126.com, suiqi0125@126.com and liangdadong@jlau.edu.cn.

*Corresponding author E-mail: sijichen@jlau.edu.cn and yjsu@jlau.edu.cn.

S1. Materials and reagents

Potatoes were bought from a local supermarket. All chemical reagents, including RhB, TC, $KCrO_7$, $(NH_4)_2SO_4$, KH_2PO_4, $MgSO_4$, $CaCl_2$, NaCl, $FeSO_4$, $MnSO_4$, $ZnCl_2$, NaOH, and HCl, were purchased from Beijing Chemical Works (Beijing, China), were of analytical grade, and they were used without further purification.

S1.2. Characterization tests

The samples were tested by using thermogravimetric analysis (TGA) and differential thermogravimetric analysis (DTG) under a nitrogen flow protection (Netzsch STA409PC, Germany). Scanning electron microscopy (SEM) was used to examine the morphology of the materials (ZEISS SIGMA HD, Germany). X-ray diffractometry (XRD) measurements were carried out using a filtered Cu-Ka X-ray source to investigate the crystalline composition of the samples (Bruker D8 Advance, Germany). A Fourier transform infrared spectrometer (FT-IR) was used to characterize the functional groups on the surface of the materials range from 400 to

4000 cm^{-1} at a resolution of 1 cm^{-1} (Thermo Fisher Nicolet iS50, USA). X-ray photoelectron spectroscopy (XPS) was used to determine the binding energy of the samples (Thermo Escalab 250Xi+, USA). A Raman spectrometer (Raman) was used to evaluate the defective of carbon samples with a wavelength of 514 nm (HORIBA LabRAM HR Evolution inVia Reflex, France). N_2 adsorption-desorption isotherms were obtained to investigate the porosity of the samples at 77 K (Quantachrome Autosorb iQ2, USA). The Brunauer-Emmett-Teller (BET) theory was used to calculate the specific surface area. The non-local density functional theory (NLDFT) and the Barrett-Joyner-Halenda (BJH) model were used to analyze the pore size distribution of the samples. A zeta potential instrument was used to characterize the surface charge of the samples (Zetasizer Nano ZS90, UK). Contact angle (CA) measurements were carried out to investigate the hydrophilicity and hydrophobicity of the samples (Dataphysics DCAT21, Germany).

S2. Results and Discussions

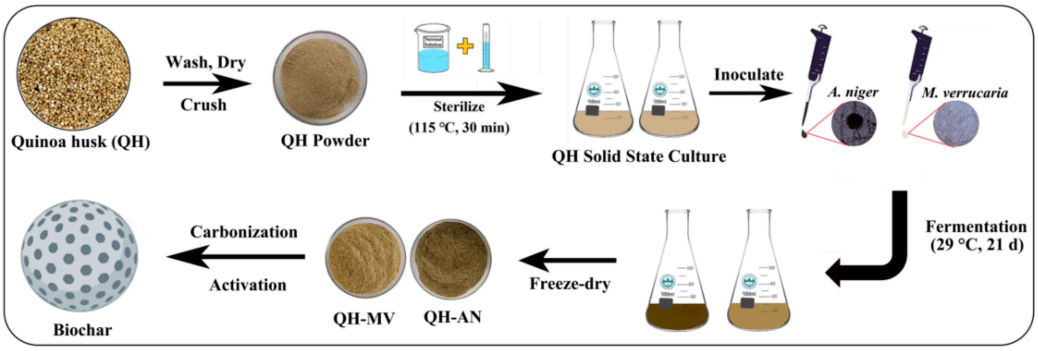

Figure S1. Scheme of the preparation of Mycelium composite biochars.

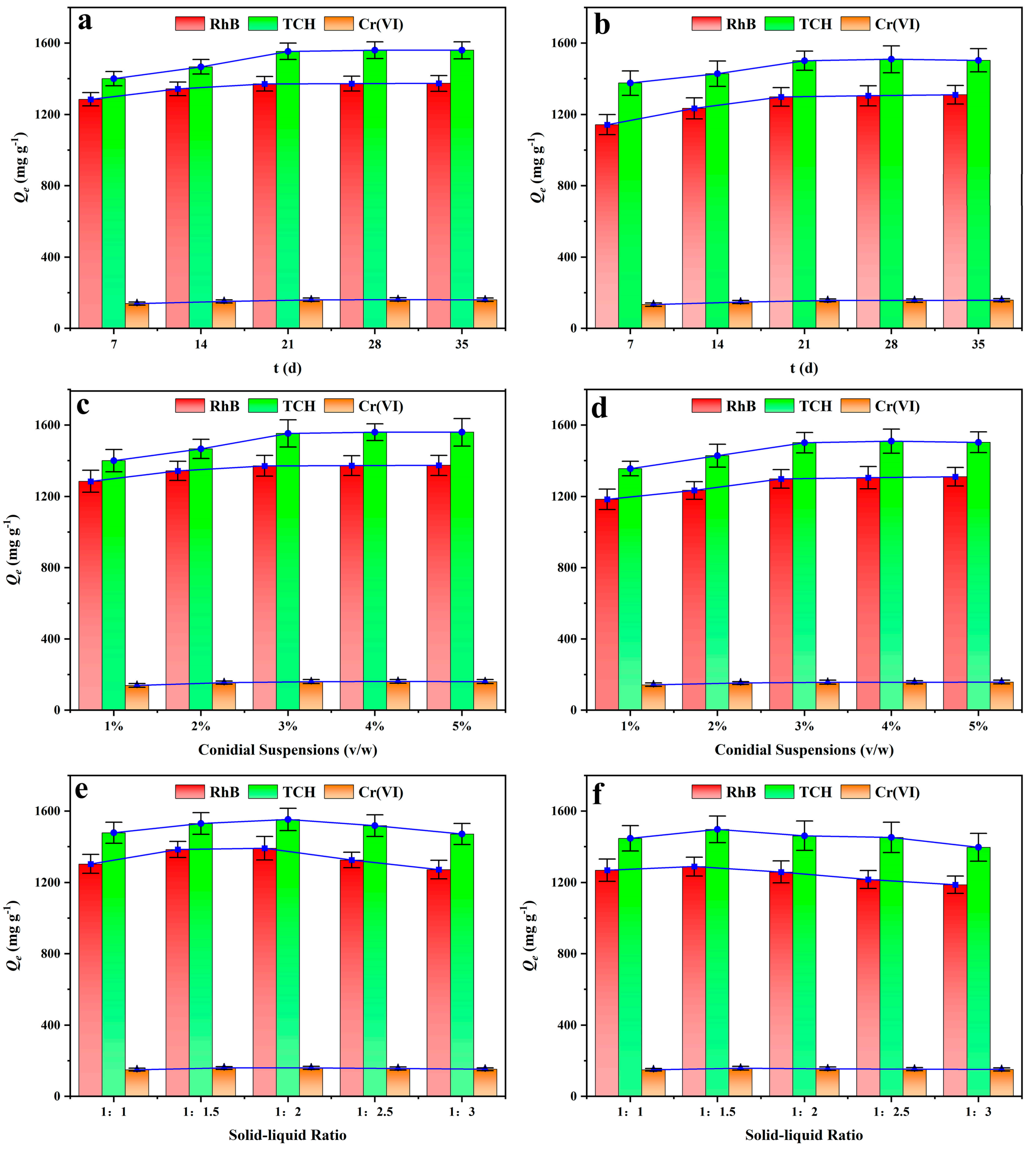

Figure S2. The optimization of the bio-regulation conditions of BQH-AN (a, c, and e) and BQH-MV (b, d, and f).

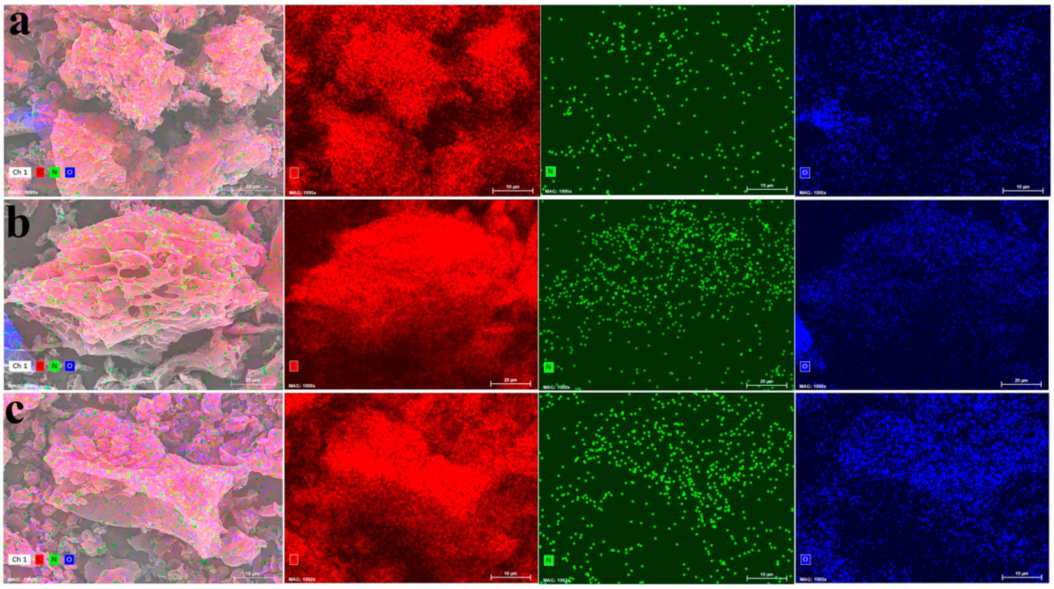

Figure S3. The EDS spectrum of BQH (a), BQH-AN (b), BQH-MV (c).

Table S1. Element analysis of Samples.

Samples	C (%)	O (%)	N (%)
QH	88.03	8.46	3.51
QH-AN	79.37	13.26	7.37
QH-MV	82.67	12.23	5.10
BQH	93.99	4.67	1.34
BQH-AN	92.69	4.33	2.98
BQH-MV	92.39	5.29	2.32

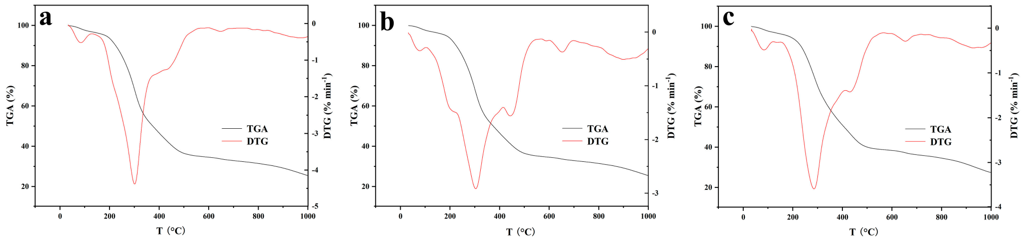

Figure S4. The TGA and DTG of QH (a), QH-AN (b), and QH-MV (c).

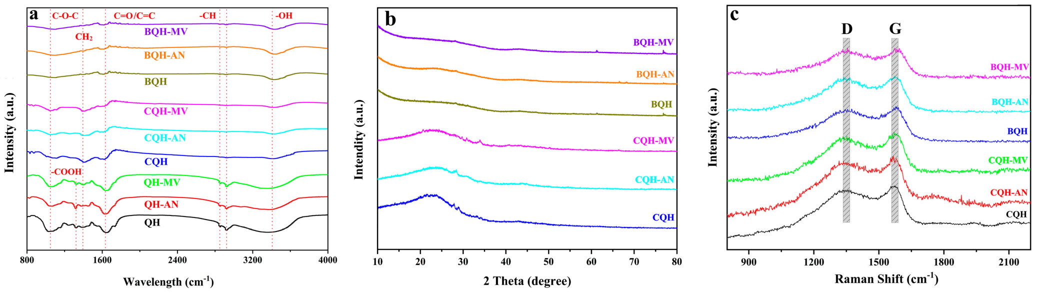

Figure S5. FT-IR spectra (a), XRD pattern (b), and Raman spectra (c) of samples.

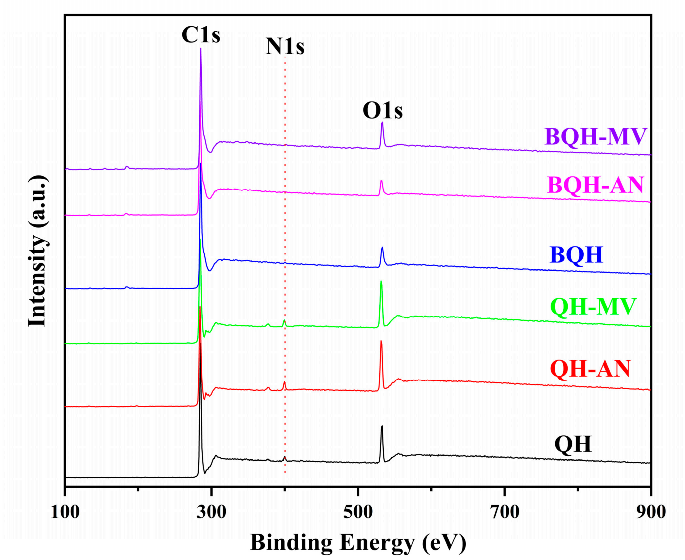

Figure S6. XPS spectra of QH, QH-AN, QH-MV, BQH, BQH-AN, and BQH-MV.

Table S2. XPS elemental composition of Samples.

Samples	C (%)	O (%)	N (%)
QH	85.67	11.82	2.51
QH-AN	78.05	16.54	5.41
QH-MV	79.35	16.42	4.23
BQH	90.80	7.97	1.23
BQH-AN	90.93	6.82	2.25
BQH-MV	88.70	9.21	2.09

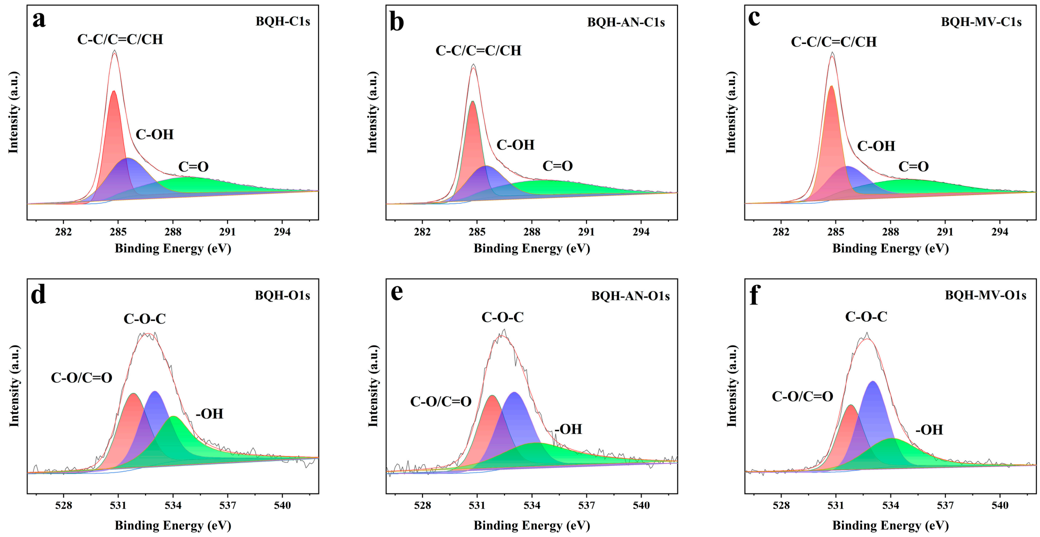

Figure S7. The C1s of the BQH (a), BQH-AN (b), BQH-MV (c); the O1s of the BQH (d), BQH-AN (e), and BQH-MV (f).

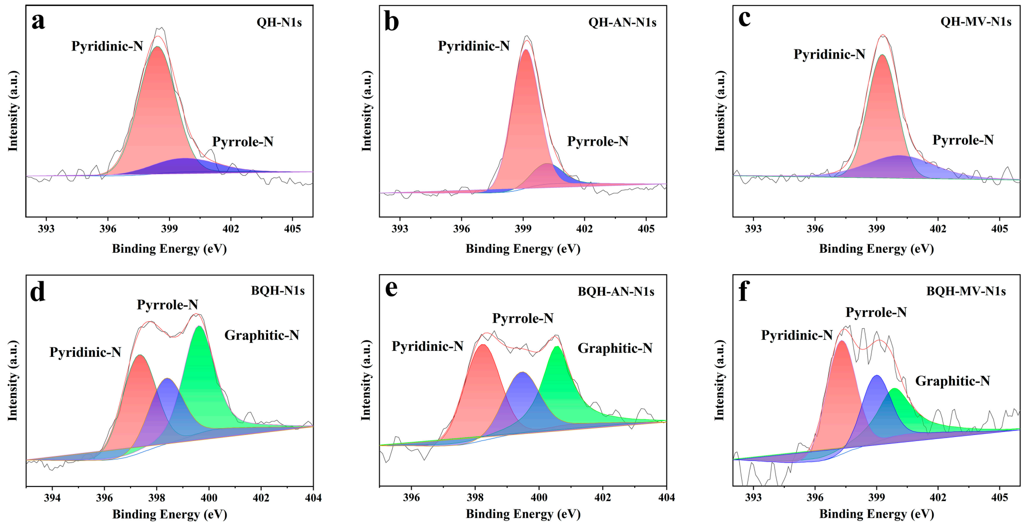

Figure S8. The N1s of the QH (a), QH-AN (b), QH-MV (c), BQH (d), BQH-AN (e), and BQH-MV (f).

Table S3. Textural data of samples obtained on the basis of N_2 adsorption-desorption.

Samples	Conditions			S_{BET}	V_{total}	P_m
	KOH:NaOH:Carbon	T (°C)	t (min)	(m^2 g^{-1})	(cm^3 g^{-1})	(nm)
CQH	-	600	60	4.78	0.0072	4.03

CQH-AN	-	600	60	19.32	0.0399	3.72
CQH-MV	-	600	60	11.58	0.0417	3.97
BQH	2 : 2 : 1	700	60	2641.31	1.81	2.74
BQH-AN	1.5 : 1.5 : 1	700	60	3547.47	2.37	2.67
BQH-MV	1.5 : 1.5 : 1	700	60	3205.59	2.46	3.07

The BET surface area, S_{BET}, in m^2 g^{-1}; Total pore volume, V_{total}, in cm^3 g^{-1}; Mean pore size, P_m, in nm.

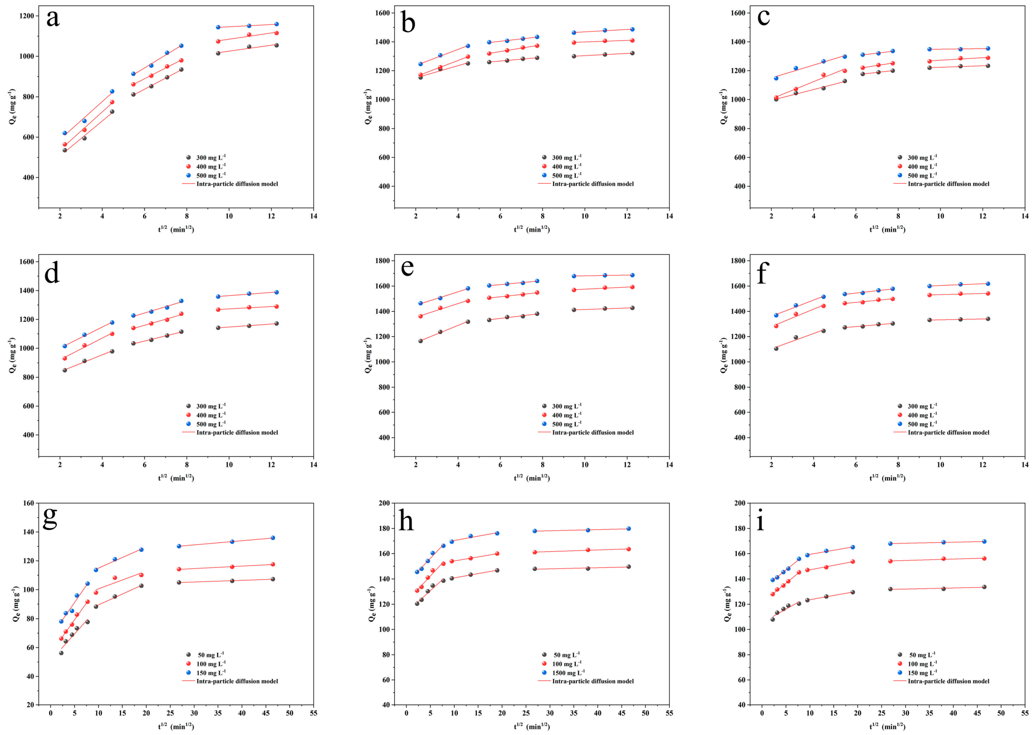

Figure S9. the Intra-particle diffusion model plots of PCQS, PCCQS-AN, and PCCQS-MV for RhB (a-c), TC (d-f), and Cr(VI) (g-i) at 303 K.

Table S4 Fitting parameters of adsorption Kinetic models for RhB at 303 K.

Sample	Kinetic models	Parameter	Dye concentration C_0 (mg L^{-1})		
			300	400	500
		Q_e (mg g^{-1})	1054.68	1115.10	1158.93
BQH	Pseudo-first-order	k_1 (min^{-1})	0.0649	0.0662	0.0751

		R^2	0.88	0.87	0.87
		SD (mg g^{-1})	113.91	120.25	122.14
		BIC	106.88	108.07	108.41
	Pseudo-second-order	k_2 (g mg^{-1} min^{-1})	0.0001	0.0001	0.0001
		$Q_{e.cat}$ (mg g^{-1})	1061.12	1119.91	1170.73
		R^2	0.98	0.98	0.98
		SD (mg g^{-1})	51.13	52.26	55.17
		BIC	89.26	89.74	90.93
	Intra-particle diffusion	k_3 (mg g^{-1} min$^{-0.5}$)	86.62	53.26	15.36
		C	333.59	569.38	1090.98
		R^2	0.97-0.98	0.98-0.99	0.92-0.99
	Bangham	k_4 (mg g^{-1} min$^{-1/n}$)	0.2737	0.2783	0.2987
		n	0.4936	0.5251	0.5007
		R^2	0.99	0.99	0.99
		SD (mg g^{-1})	31.78	30.23	34.68
		BIC	78.58	77.69	80.72
BQH-AN		Parameter	Dye concentration C_0 (mg L^{-1})		
			300	400	500
		Q_e (mg g^{-1})	1321.83	1409.80	1486.55
	Pseudo-first-order	k_1 (min^{-1})	0.3641	0.3141	0.3387
		R^2	0.99	0.97	0.98
		SD (mg g^{-1})	49.09	71.25	75.62
		BIC	88.36	96.56	97.86
	Pseudo-second-order	k_2 (g mg^{-1} min^{-1})	0.0011	0.0006	0.007

		$Q_{e.cat}$ (mg g^{-1})	1336.12	1427.32	1501.24
		R^2	0.99	0.99	0.99
		SD (mg g^{-1})	12.42	21.92	23.15
		BIC	58.13	70.63	71.82
	Intra-particle diffusion	k_3 (mg g^{-1} min$^{-0.5}$)	42.48	23.95	16.55
		C	1065.33	1189.26	1304.88
		R^2	0.95-0.99	0.99-0.99	0.90-0.99
	Bangham	k_4 (mg g^{-1} min$^{-1/n}$)	1.328	1.1495	1.6374
		n	0.3174	0.2794	0.2856
		R^2	0.99	0.99	0.99
		SD (mg g^{-1})	5.98	10.39	11.02
		BIC	42.03	54.21	55.49

		Parameter	Dye concentration C_0 (mg L^{-1})		
			300	400	500
BQH-MV		Q_e (mg g^{-1})	1233.96	1289.47	1354.68
	Pseudo-first-order	k_1 (min^{-1})	0.2845	0.2604	0.3444
		R^2	0.95	0.96	0.98
		SD (mg g^{-1})	79.88	73.74	52.01
		BIC	99.07	97.31	89.63
	Pseudo-second-order	k_2 (g mg^{-1} min^{-1})	0.0004	0.0005	0.0008
		$Q_{e.cat}$ (mg g^{-1})	1237.26	1285.47	1350.46
		R^2	0.99	0.99	0.99
		SD (mg g^{-1})	32.72	21.96	13.65
		BIC	79.43	70.66	60.19

	Intra-particle diffusion	k_3 (mg g^{-1} min$^{-0.5}$)	59.70	22.46	17.68
		C	885.90	1079.25	1198.09
		R^2	0.96-0.98	0.95-0.99	0.89-0.97
	Bangham	k_4 (mg g^{-1} min$^{-1/n}$)	0.9734	0.8713	1.1400
		n	0.2985	0.3351	0.3042
		R^2	0.99	0.99	0.99
		SD (mg g^{-1})	20.86	10.77	6.49
		BIC	69.53	54.98	43.86

Table S5 Fitting parameters of adsorption Kinetic models for TC at 303 K.

Sample	Kinetic models	Parameter	Antibiotic concentration C_0		
			300	400	500
		Q_e (mg g^{-1})	1171.59	1289.55	1388.71
BQH	Pseudo-first-order	k_1 (min^{-1})	0.1973	0.2026	0.2050
		R^2	0.91	0.93	0.92
		SD (mg g^{-1})	106.87	102.64	116.36
		BIC	105.20	104.59	109.35
	Pseudo-second-order	k_2 (g mg^{-1} min^{-1})	0.0003	0.0004	0.0004
		$Q_{e.cat}$ (mg g^{-1})	1173.26	1296.47	1397.62
		R^2	0.99	0.99	0.99
		SD (mg g^{-1})	35.66	35.78	41.86
		BIC	81.33	81.40	84.85
	Intra-particle diffusion	k_3 (mg g^{-1} min$^{-0.5}$)	58.38	42.16	11.21
		C	721.51	906.89	1252.93

		R^2	0.98-0.99	0.96-0.99	0.92-0.99
	Bangham	k_4 (mg g^{-1}	0.7079	0.7043	0.7316
		n	0.3384	0.3494	0.3336
		R^2	0.99	0.99	0.99
		SD (mg g^{-1})	17.85	18.91	21.91
		BIC	66.10	67.37	70.61

		Parameter	Antibiotic concentration C_0		
			300	400	500
BQH-AN		Q_e (mg g^{-1})	1426.60	1592.12	1687.04
	Pseudo-first-order	k_1 (min^{-1})	0.2994	0.3076	0.3671
		R^2	0.96	0.97	0.98
		SD (mg g^{-1})	74.23	73.83	70.31
		BIC	97.16	96.42	95.48
	Pseudo-second-order	k_2 (g mg^{-1} min^{-1})	0.0006	0.007	0.0007
		$Q_{e.cat}$ (mg g^{-1})	1410.12	1597.26	1699.85
		R^2	0.99	0.99	0.99
		SD (mg g^{-1})	21.46	20.95	25.63
		BIC	70.16	69.63	74.07
	Intra-particle diffusion	k_3 (mg g^{-1} min$^{-0.5}$)	63.07	18.28	15.67
		C	1016.55	1405.66	1517.51
		R^2	0.98-0.99	0.98-0.99	0.96-0.98
	Bangham	k_4 (mg g^{-1}	1.0420	1.2580	1.3007
		n	0.2943	0.2486	0.2535
		R^2	0.99	0.99	0.99

	SD (mg g^{-1})	10.80	9.68	14.44
	BIC	55.06	52.64	61.44

		Parameter	Antibiotic concentration C_0 [illegible]		
			300	400	500
		Q_e (mg g^{-1})	1340.36	1540.64	1618.68
BQH-MV	Pseudo-first-order	k_1 (min^{-1})	0.3166	0.3275	0.3402
		R^2	0.98	0.97	0.98
		SD (mg g^{-1})	57.10	68.41	61.71
		BIC	91.68	95.99	93.76
	Pseudo-second-order	k_2 (g mg^{-1} min^{-1})	0.0007	0.0006	0.0007
		$Q_{e.cat}$ (mg g^{-1})	1352.46	1548.24	1631.78
		R^2	0.99	0.99	0.99
		SD (mg g^{-1})	14.07	16.11	18.38
		BIC	60.87	63.85	66.75
	Intra-particle diffusion	k_3 (mg g^{-1} $min^{-0.5}$)	60.93	18.92	14.21
		C	981.19	1430.93	1194.09
		R^2	0.93-0.97	0.95-0.97	0.92-0.99
	Bangham	k_4 (mg g^{-1}	1.0706	1.1167	1.1871
		n	0.3051	0.2869	0.2775
		R^2	0.99	0.99	0.99
		SD (mg g^{-1})	7.61	9.16	8.45
		BIC	47.36	51.43	49.65

Table S6 Fitting parameters of adsorption Kinetic models for Cr(VI) at 303 K.

Sample	Kinetic models	Parameter	Cr(VI) concentration C_0 (mg L^{-1})		
			50	100	150
		Q_e (mg g^{-1})	107.09	117.57	135.21
BQH	Pseudo-first-order	k_1 (min^{-1})	0.0645	0.0715	0.0733
		R^2	0.74	0.75	0.73
		SD (mg g^{-1})	17.71	16.55	21.04
		BIC	65.65	63.73	69.72
	Pseudo-second-order	k_2 (g mg^{-1} min^{-1})	0.0016	0.0016	0.0015
		$Q_{e.cat}$ (mg g^{-1})	106.41	115.21	137.85
		R^2	0.93	0.94	0.93
		SD (mg g^{-1})	9.26	8.47	10.40
		BIC	51.86	49.71	54.22
	Intra-particle diffusion	k_3 (mg g^{-1} $min^{-0.5}$)	3.71	1.23	1.46
		C	50.92	88.37	100.40
		R^2	0.92-0.99	0.91-0.99	0.97-0.99
	Bangham	k_4 (mg g^{-1}	0.4163	0.4508	0.4738
		n	0.3241	0.3184	0.2769
		R^2	0.99	0.99	0.99
		SD (mg g^{-1})	3.03	2.86	3.88
		BIC	27.10	25.82	32.55
		Parameter	Cr(VI) concentration C_0 (mg L^{-1})		
			50	100	150
		Q_e (mg g^{-1})	149.92	163.42	179.76
BQH-AN	Pseudo-first-order	k_1 (min^{-1})	0.2666	0.2714	0.2865

		R^2	0.94	0.93	0.92
		SD (mg g^{-1})	11.56	12.88	14.57
		BIC	56.55	58.92	61.63
	Pseudo-second-order	k_2 (g mg^{-1} min^{-1})	0.0052	0.0048	0.0047
		$Q_{e.cat}$ (mg g^{-1})	144.51	165.38	182.49
		R^2	0.99	0.99	0.99
		SD (mg g^{-1})	4.42	5.28	6.43
		BIC	35.40	39.30	43.64
	Intra-particle diffusion	k_3 (mg g^{-1} min$^{-0.5}$)	3.45	1.63	1.12
		C	113.53	147.86	157.77
		R^2	0.95-0.97	0.91-0.99	0.87-0.98
	Bangham	k_4 (mg g^{-1}	1.1287	1.1726	1.1198
		n	0.2047	0.1954	0.2028
		R^2	0.99	0.99	0.99
		SD (mg g^{-1})	1.20	1.91	2.62
		BIC	6.63	16.95	23.86

		Parameter	Cr(VI) concentration C_0 (mg L^{-1})		
			50	100	150
BQH-MV		Q_e (mg g^{-1})	133.07	156.06	168.55
	Pseudo-first-order	k_1 (min^{-1})	0.2855	0.2891	0.2925
		R^2	0.95	0.94	0.91
		SD (mg g^{-1})	10.43	12.09	13.50
		BIC	54.28	57.54	59.96
	Pseudo-second-order	k_2 (g mg^{-1} min^{-1})	0.0070	0.0056	0.0053

	$Q_{e.cat}$ (mg g^{-1})	127.58	159.84	167.37
	R^2	0.99	0.99	0.99
	SD (mg g^{-1})	4.13	5.08	5.91
	BIC	33.90	38.44	41.80
Intra-particle diffusion	k_3 (mg g^{-1} min$^{-0.5}$)	2.13	1.67	1.18
	C	105.44	116.85	129.51
	R^2	0.86-0.98	0.98-0.99	0.93-0.99
Bangham	k_4 (mg g^{-1}	1.2007	1.2044	1.2196
	n	0.1948	0.1847	0.1774
	R^2	0.99	0.99	0.99
	SD (mg g^{-1})	1.40	1.77	2.25
	BIC	10.09	15.24	20.51

Table S7 Fitting parameters of adsorption isotherm models for RhB.

Sample	Isotherm types	Constants			
			293 K	303 K	313 K
BQH	Langmuir	Q_m (mg g^{-1})	1104.81	1165.17	1228.74
		K_L (L mg^{-1})	0.0438	0.0614	0.0454
		R^2	0.94	0.94	0.93
		SD (mg g^{-1})	16.92	17.46	17.72
		BIC	64.93	65.62	65.94
	Freundlich	K_F (mg g^{-1}(L	461.00	534.23	605.46
		n_F	7.17	7.98	8.74
		R^2	0.99	0.99	0.99

		SD (mg g^{-1})	3.87	1.99	1.61
		BIC	32.44	17.89	13.13
	Temkin	A (L g^{-1})	8.36	19.360	43.33
		b	19.92	20.67	21.18
		R^2	0.99	0.99	0.99
		SD (mg g^{-1})	4.79	3.80	3.32
		BIC	37.14	32.04	29.07
	Liu	Kg（L mg^{-1}）	0.0454	0.0551	0.0654
		n_L	1.1021	1.0553	1.0212
		R^2	0.92	0.92	0.93
		SD (mg g^{-1})	19.38	19.08	18.80
		BIC	67.91	67.57	67.24
BQH-AN	Langmuir	Q_m (mg g^{-1})	1396.24	1448.34	1483.86
		K_L (L mg^{-1})	0.1251	0.1373	0.1756
		R^2	0.90	0.87	0.90
		SD (mg g^{-1})	18.33	20.83	17.82
		BIC	66.69	69.50	66.06
	Freundlich	K_F (mg g^{-1}(L [illegible]	896.41	945.15	1038.83
		n_F	13.62	14.06	16.74
		R^2	0.99	0.99	0.99
		SD (mg g^{-1})	3.15	4.82	3.30
		BIC	27.95	37.30	28.98
	Temkin	A (L g^{-1})	6770.07	11115.84	166637.28
		b	27.74	27.57	31.59

		R^2	0.99	0.98	0.99
		SD (mg g^{-1})	4.47	6.22	4.32
		BIC	35.64	42.90	34.87
	Liu	Kg (L mg^{-1})	0.1622	0.1928	0.2561
		n_L	0.8648	0.8222	0.8203
		R^2	0.91	0.90	0.91
		SD (mg g^{-1})	17.08	18.94	16.11
		BIC	65.13	67.41	63.84
BQH-MV	Langmuir	Q_m (mg g^{-1})	1298.98	1352.10	1411.63
		K_L (L mg^{-1})	0.1474	0.1755	0.2206
		R^2	0.90	0.92	0.90
		SD (mg g^{-1})	13.37	11.98	12.33
		BIC	59.74	57.34	57.97
	Freundlich	K_F (mg g^{-1}(L	899.03	976.12	1066.20
		n_F	16.46	18.51	21.32
		R^2	0.99	0.99	0.99
		SD (mg g^{-1})	1.68	1.27	2.32
		BIC	14.16	7.94	21.17
	Temkin	A (L g^{-1})	110033.45	872262.25	1550700.27
		b	35.43	37.96	41.58
		R^2	0.99	0.99	0.99
		SD (mg g^{-1})	2.52	1.62	2.64
		BIC	23.06	13.36	24.03
	Liu	Kg（L mg^{-1}）	0.1934	0.2236	0.3061

		n_L	0.8744	0.8968	0.8661
		R^2	0.92	0.93	0.92
		SD (mg g^{-1})	12.50	11.39	11.52
		BIC	58.27	56.22	56.46

Table S8 Fitting parameters of adsorption isotherm models for TC.

Sample	Isotherm types	Constants			
			293 K	303 K	313 K
BQH	Langmuir	Q_m (mg g^{-1})	1205.02	1329.59	1366.99
		K_L (L mg^{-1})	0.0567	0.0607	0.0792
		R^2	0.93	0.92	0.92
		SD (mg g^{-1})	18.73	21.93	20.43
		BIC	67.16	70.63	69.08
	Freundlich	K_F (mg g^{-1}(L $^{1)}$ $^{1/n)}$	570.30	633.91	737.65
		n_F	8.29	8.31	9.09
		R^2	0.99	0.99	0.99
		SD (mg g^{-1})	2.36	2.27	1.87
		BIC	21.60	20.73	16.46
	Temkin	A (L g^{-1})	27.45	29.74	151.81
		b	20.65	18.79	21.26
		R^2	0.99	0.99	0.99
		SD (mg g^{-1})	4.58	5.10	4.14
		BIC	36.16	38.56	33.95
	Liu	Kg（L mg^{-1}）	0.0614	0.0667	0.0901

		n_L	0.9984	0.9823	0.9418
		R^2	0.92	0.91	0.94
		SD (mg g^{-1})	19.40	22.46	16.18
		BIC	67.94	71.15	58.80
BQH-AN	Langmuir	Q_m (mg g^{-1})	1575.33	1642.35	1666.86
		K_L (L mg^{-1})	0.1301	0.1465	0.2748
		R^2	0.91	0.91	0.90
		SD (mg g^{-1})	21.11	21.66	22.98
		BIC	70.81	71.35	79.68
	Freundlich	K_F (mg g^{-1}(L	985.88	1052.99	1247.57
		n_F	11.28	13.37	20.16
		R^2	0.99	0.99	0.99
		SD (mg g^{-1})	1.93	3.08	6.10
		BIC	17.16	27.45	42.48
	Temkin	A (L g^{-1})	3081.42	5949.53	6313788.91
		b	23.18	23.21	33.58
		R^2	0.99	0.99	0.98
		SD (mg g^{-1})	3.88	4.20	7.14
		BIC	32.53	34.25	45.96
	Liu	Kg（L mg^{-1}）	0.1706	0.1952	0.5712
		n_L	0.8417	0.8357	0.6964
		R^2	0.93	0.93	0.89
		SD (mg g^{-1})	19.03	18.42	20.57
		BIC	66.64	66.06	69.97

	Langmuir	Q_m (mg g^{-1})	1464.41	1536.32	1578.93
		K_L (L mg^{-1})	0.1017	0.1123	0.1324
		R^2	0.89	0.89	0.88
		SD (mg g^{-1})	23.93	24.86	25.46
		BIC	72.55	73.25	75.92
	Freundlich	K_F (mg g^{-1}(L	854.70	914.69	987.80
		n_F	11.21	11.57	12.72
		R^2	0.99	0.99	0.99
		SD (mg g^{-1})	5.51	5.61	6.19
BQH-MV		BIC	40.22	40.63	42.79
	Temkin	A (L g^{-1})	620.16	934.51	3071.08
		b	22.21	21.80	23.11
		R^2	0.99	0.99	0.99
		SD (mg g^{-1})	7.32	7.67	7.84
		BIC	46.50	47.51	48.00
	Liu	Kg（L mg^{-1}）	0.1262	0.1454	0.1778
		n_L	0.8778	0.8475	0.8332
		R^2	0.90	0.90	0.91
		SD (mg g^{-1})	22.61	22.89	21.84
		BIC	72.31	72.51	71.00

Table S9 Fitting parameters of adsorption isotherm models for Cr(VI).

Sample	Isotherm types	Constants			
			293 K	303 K	313 K

BQH	Langmuir	Q_m (mg g^{-1})	144.69	148.07	166.29
		K_L (L mg^{-1})	0.0352	0.0387	0.0345
		R^2	0.99	0.99	0.99
		SD (mg g^{-1})	0.95	0.96	1.07
		BIC	1.66	1.86	4.16
	Freundlich	K_F (mg g^{-1}(L	27.80	31.69	31.00
		n_F	3.29	3.51	3.23
		R^2	0.98	0.97	0.97
		SD (mg g^{-1})	1.27	1.64	1.79
		BIC	7.95	13.64	15.50
	Temkin	*A* (L g^{-1})	0.4078	0.4988	0.3828
		b	85.52	86.32	73.45
		R^2	0.99	0.98	0.99
		SD (mg g^{-1})	0.96	1.27	1.21
		BIC	1.85	7.84	6.91
	Liu	*Kg*（L mg^{-1}）	0.0398	0.0403	0.0391
		n_L	2.2341	2.3484	2.3321
		R^2	0.95	0.97	0.97
		SD (mg g^{-1})	1.99	1.47	1.52
		BIC	17.89	11.12	13.15
BQH-AN	Langmuir	Q_m (mg g^{-1})	188.50	205.42	217.24
		K_L (L mg^{-1})	0.0398	0.0415	0.0419
		R^2	0.99	0.99	0.99
		SD (mg g^{-1})	1.34	0.81	1.48

		BIC	9.19	1.19	11.35
	Freundlich	K_F (mg g^{-1}(L	40.89	46.03	49.21
		n_F	3.53	3.59	3.62
		R^2	0.95	0.97	0.95
		SD (mg g^{-1})	2.62	2.29	3.15
		BIC	23.88	20.92	27.96
	Temkin	*A* (L g^{-1})	0.5129	0.5605	0.5665
		b	67.84	63.12	59.71
		R^2	0.97	0.98	0.97
		SD (mg g^{-1})	2.07	1.68	2.49
		BIC	18.72	14.08	22.77
	Liu	*Kg*（L mg^{-1}）	0.0408	0.0406	0.0418
		n_L	2.4151	2.2915	2.4905
		R^2	0.98	0.97	0.98
		SD (mg g^{-1})	1.64	2.06	1.91
		BIC	13.64	18.60	16.88
BQH-MV	Langmuir	Q_m (mg g^{-1})	175.99	186.84	189.31
		K_L (L mg^{-1})	0.0484	0.0526	0.0712
		R^2	0.99	0.99	0.99
		SD (mg g^{-1})	0.90	0.91	0.92
		BIC	0.48	0.67	0.91
	Freundlich	K_F (mg g^{-1}(L	46.5589	53.1490	70.2836
		n_F	4.0231	4.2398	5.3415
		R^2	0.98	0.98	0.95

	SD (mg g^{-1})	1.46	1.48	2.16
	BIC	11.04	11.37	19.69
Temkin	*A* (L g^{-1})	0.8684	1.0870	3.1795
	b	79.4464	77.5069	90.0669
	R^2	0.99	0.99	0.98
	SD (mg g^{-1})	1.07	1.08	1.86
	BIC	4.29	4.36	16.37
Liu	*Kg*（L mg^{-1}）	0.0464	0.0484	0.0522
	n_L	2.1896	2.1643	2.3514
	R^2	0.95	0.96	0.98
	SD (mg g^{-1})	2.20	2.16	1.14
	BIC	20.09	19.68	5.65

Table S10. Thermodynamic parameters for the adsorption of RhB onto BQH, BQH-AN, and BQH-MV.

Samples	Temperature (K)	ΔG (kJ mol^{-1})	ΔH (kJ mol^{-1})	ΔS (J mol^{-1} K^{-1})
BQH	293	-2.63		
	303	-2.96	33.21	9.73
	313	-3.18		
BQH-AN	293	-3.57		
	303	-3.82	24.77	7.25
	313	-4.04		
BQH-MV	293	-3.13		
	303	-3.39	26.01	7.61
	313	-3.59		

Table S11. Thermodynamic parameters for the adsorption of TC onto BQH, BQH-AN,

and BQH-MV.

Samples	Temperature (K)	ΔG (kJ mol^{-1})	ΔH (kJ mol^{-1})	ΔS (J mol^{-1} K^{-1})
BQH	293	-3.04		
	303	-3.39	35.22	10.32
	313	-3.64		
BQH-AN	293	-3.78		
	303	-4.04	25.68	7.52
	313	-4.37		
BQH-MV	293	-3.68		
	303	-3.94	25.25	7.39
	313	-4.16		

Table S12. Thermodynamic parameters for the adsorption of Cr(VI) onto BQH, BQH-AN, and BQH-MV.

Samples	Temperature (K)	ΔG (kJ mol^{-1})	ΔH (kJ mol^{-1})	ΔS (J mol^{-1} K^{-1})
BQH	293	-0.30		
	303	-0.56	26.19	7.67
	313	-0.87		
BQH-AN	293	-1.21		
	303	-1.45	23.98	7.03
	313	-1.80		
BQH-MV	293	-1.06		
	303	-1.33	26.33	7.71
	313	-1.70		

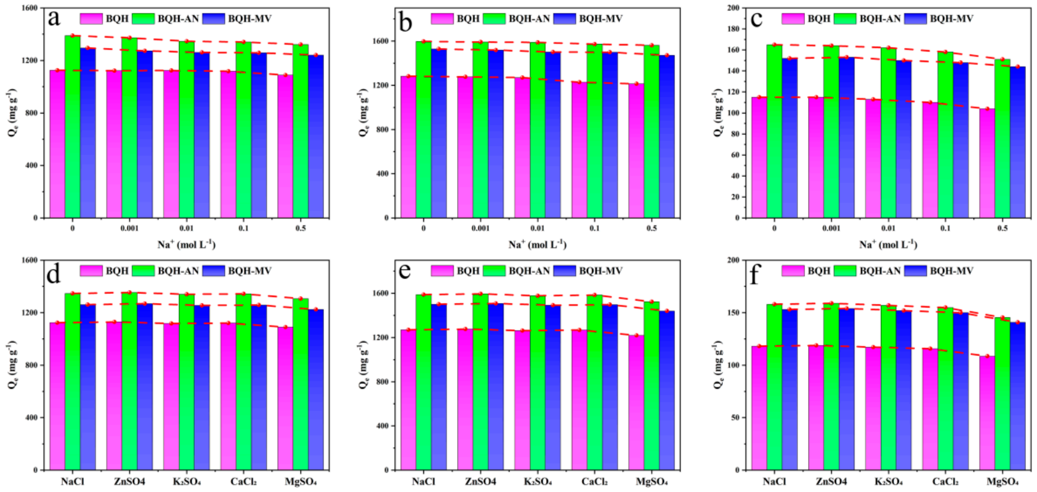

Figure S10. Effect of coexisting ions on RhB (a, d), TC (b, e), and Cr(VI) (c, f) adsorption.

Table S13 Comparison of the adsorption capacities of samples to RhB with other adsorbents.

Adsorbent	pH	T (K)	C_0 (mg L^{-1})	Q_e (mg g^{-1})	References
Fe-N-BC	7	298	200	12.41	[S1]
ASC	7	298	100	123.46	[S2]
Activated carbon derived from carbon residue from biomass gasification	11	298	232	190.21	[S3]
Hierarchical SnS_2 nanostructure	-	298	100	200.47	[S4]
Functionalized graphene via tannic acid	11	318	256	201.13	[S5]
waste carton mesoporous biochar	7.33	303	25	187.40	[S6]
Tannery residual biomass (TRB)	3	30	200	250.18	[S7]

		3			
Gelatin/activated carbon composite beads	6	303	200	256.19	[S8]
Activated carbon prepared from bagasse pith	2	343	600	264.48	[S9]
Polymer modified biomass of baker's yeast	11	286	50	267.94	[S10]
Magnetic AC/CeO_2	7	293	50	325.86	[S11]
CCAP	11	273	200	374.30	[S12]
Pyruvic acid (PA)-modified activated carbons	7	295	100	385.76	[S13]
N-vinylimidazole modified hyper-cross-linked resins	7	318	100	421.84	[S14]
Oil palm empty fruit bunches activated carbon	7	308	180	982.87	[S15]
Nitrogen-doped porous cork activated carbon	-	308	200	996.75	[S16]
Honeycomb-like cork activated carbon	6	308	1600	1730.12	[S17]
UiO-66-$(COOH)_2$	7	303	3000	2200.00	[S18]
BQH	5	303	400	1134.78	This work
BQH-AN	5	303	400	1450.79	This work
BQH-MV	5	303	400	1329.85	This work

Table S14 Comparison of the adsorption capacities of samples to TC with other adsorbents.

Adsorbent	pH	T (K)	C_0 (mg L^{-1})	Q_e (mg g^{-1})	References
Rice husk ash	5	298	50	8.37	[S19]
Red earth clay composite	9	303	20	14.90	[S20]
Grapefruit peel	7	318	50	32.50	[S21]
Poplar saw dust	5	298	250	60.90	[S22]
BCFS800	7	293	100	60.70	[S23]
BM-biochars	6-8	298	25	84.54	[S24]
Chicken bone	8	298	100	98.90	[S25]
Municipal sludge	7	308	200	121.80	[S26]
MGO	5	313	50	141.44	[S27]
x-mAC	8	298	100	221.73	[S28]
Graphene oxide	3	298	166	313.55	[S29]
NaOH-activated carbon	3	30	600	455.33	[S30]

		3			
Magnetic carbon-coated cobalt oxide nanoparticles	8	303	200	769.00	[S31]
SG-ELBC	6	298	300	1163.00	[S32]
MIL-53 (Fe)	9	298	200	1250.00	[S33]
BQH	5	303	400	1280.88	This work
BQH-AN	5	303	400	1608.43	This work
BQH-MV	5	303	400	1526.46	This work

Table S15 Comparison of the adsorption capacities of samples to Cr(VI) with other adsorbents.

Adsorbent	pH	T (K)	C_0 (mg L^{-1})	Q_e (mg g^{-1})	References
CMPBC	3.0	298	5	14.60	[S34]
CMLB	3.0	-	40	30.14	[S35]
Ch-ASC400	1.5	303	55	37.48	[S36]
OCS-160	3.0	-	100	83.60	[S37]
FSBC(1:1:1)	4.	31	-	101.3	[S38]

	5	3		8	
Mesoporous carbon-doped Al_2O_3 adsorbent	4.0	318	100	114.42	[S39]
Woody-activated carbon	2.0	303	100	154.56	[S40]
HCl pretreated Elm tree sawdust	2.0	-	50	190	[S41]
Glucose	2.0	-	200	230.2	[S42]
NCDs-CNF/CSgel	2.0	RT	-	294.46	[S43]
lignin-based carbon materials	2.0	318	250	326	[S44]
N, S co-doped porous carbon	2.0	298	100	357.57	[S45]
BQH	2.0	303	100	701.33	This work
BQH-AN	2.0	303	100	744.15	This work
BQH-MV	2.0	303	100	752.27	This work

Table S16. Textural data of samples obtained on the basis of N_2 adsorption-desorption.

Samples	S_{BET} (m^2 g^{-1})	V_{total} (cm^3 g^{-1})
BQH	2641.31	1.81
BQH-RhB	549.32	0.45
BQH-TC	1678.47	0.89

BQH-Cr(VI)	321.20	0.26
BQH-AN	3547.47	2.37
BQH-AN-RhB	553.36	0.44
BQH-AN-TC	1547.62	0.86
BQH-AN-Cr(VI)	294.07	0.25
BQH-MV	3205.59	2.46
BQH-MV-RhB	773.82	0.60
BQH-MV-TC	1620.60	0.92
BQH-MV-Cr(VI)	718.43	0.60

The BET surface area, S_{BET}, in $m^2\ g^{-1}$; Total pore volume, V_{total}, in $cm^3\ g^{-1}$.

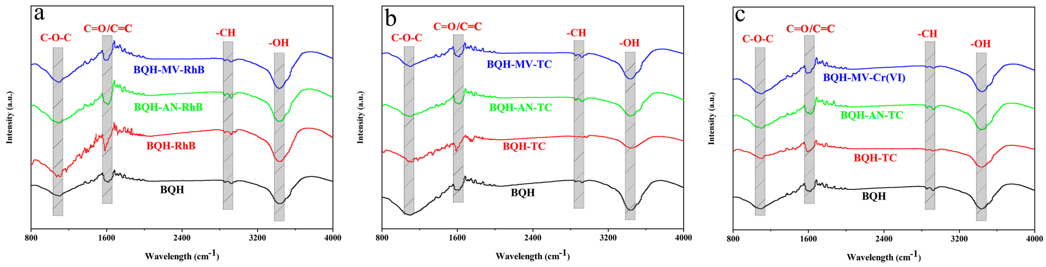

Figure S11. FT-IR spectra of samples adsorbed with RhB (a), TC (b) and Cr(VI) (c).

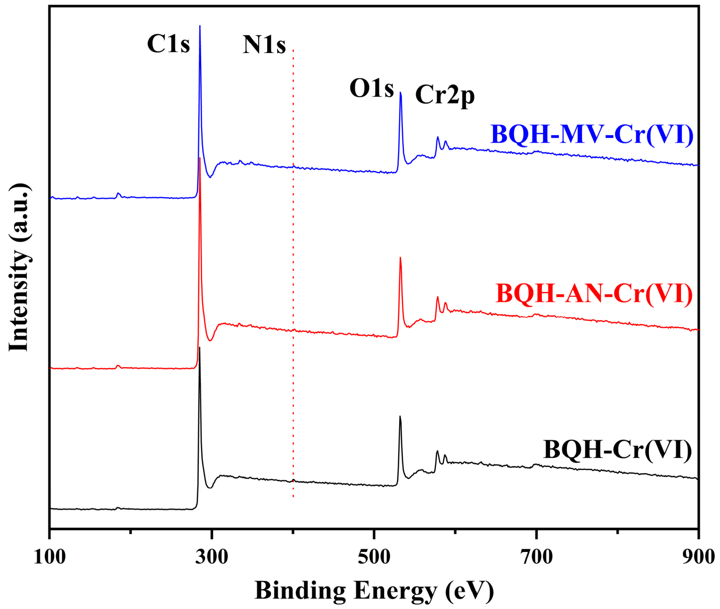

Figure S12. XPS spectra of samples adsorbed with Cr(VI).

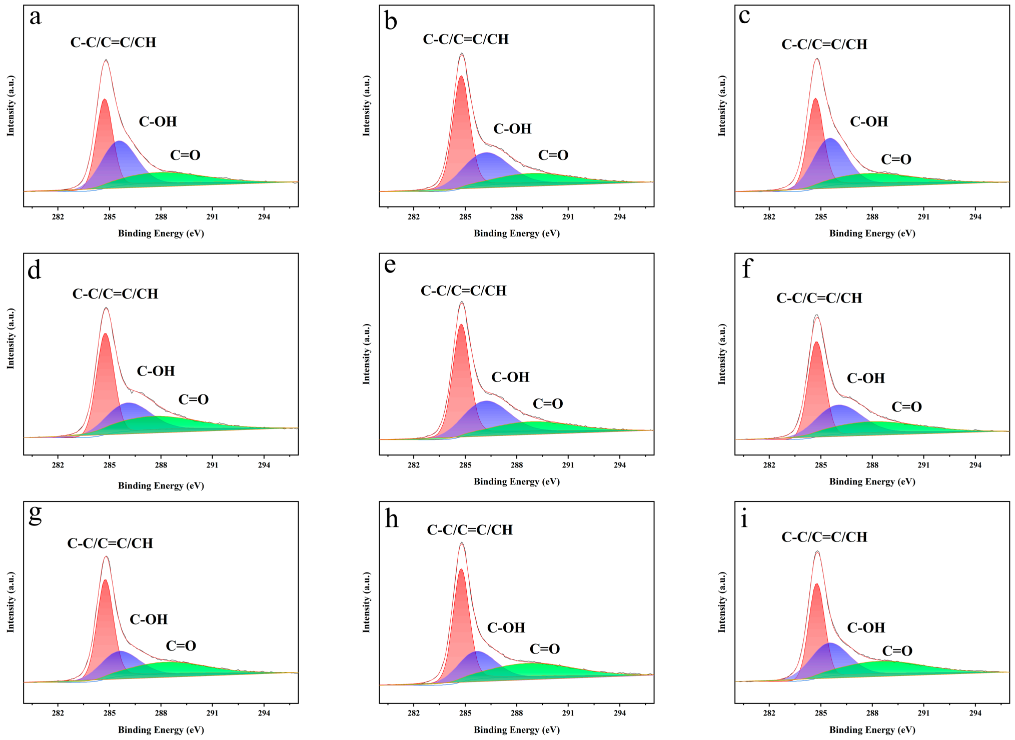

Figure S13. The C1s of the BQH, BQH-AN, BQH-MV adsorbed with RhB (a-c), TC (d-f) and Cr(VI) (g-i).

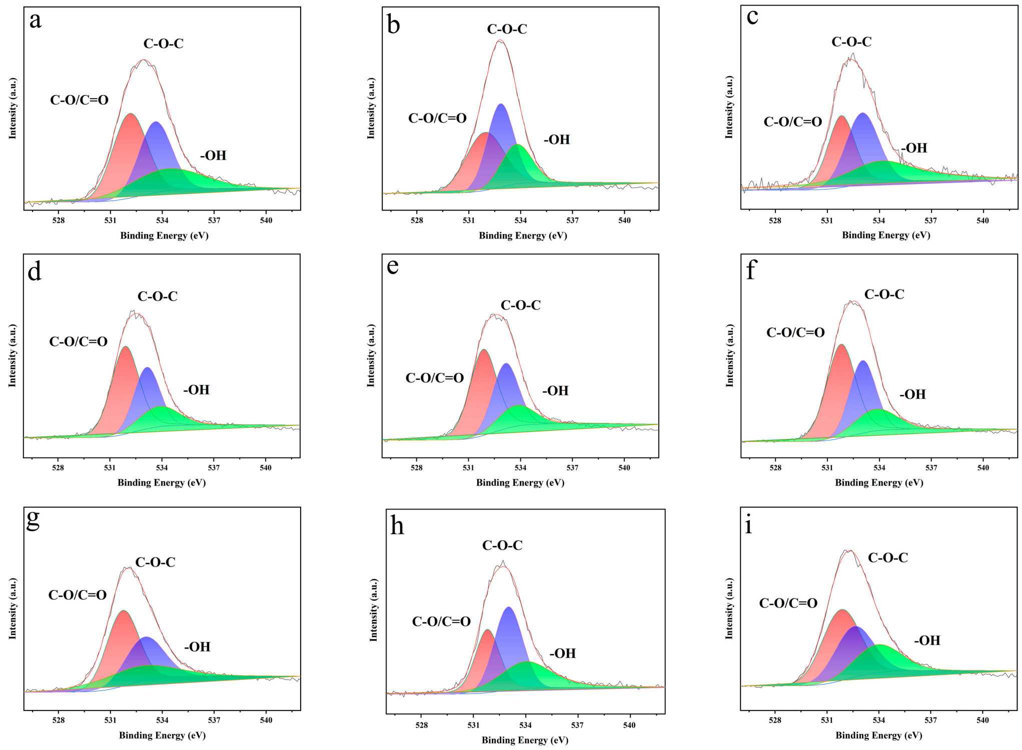

Figure S14. The O1s of the BQH, BQH-AN, BQH-MV adsorbed with RhB (a-c), TC (d-f) and Cr(VI) (g-i).

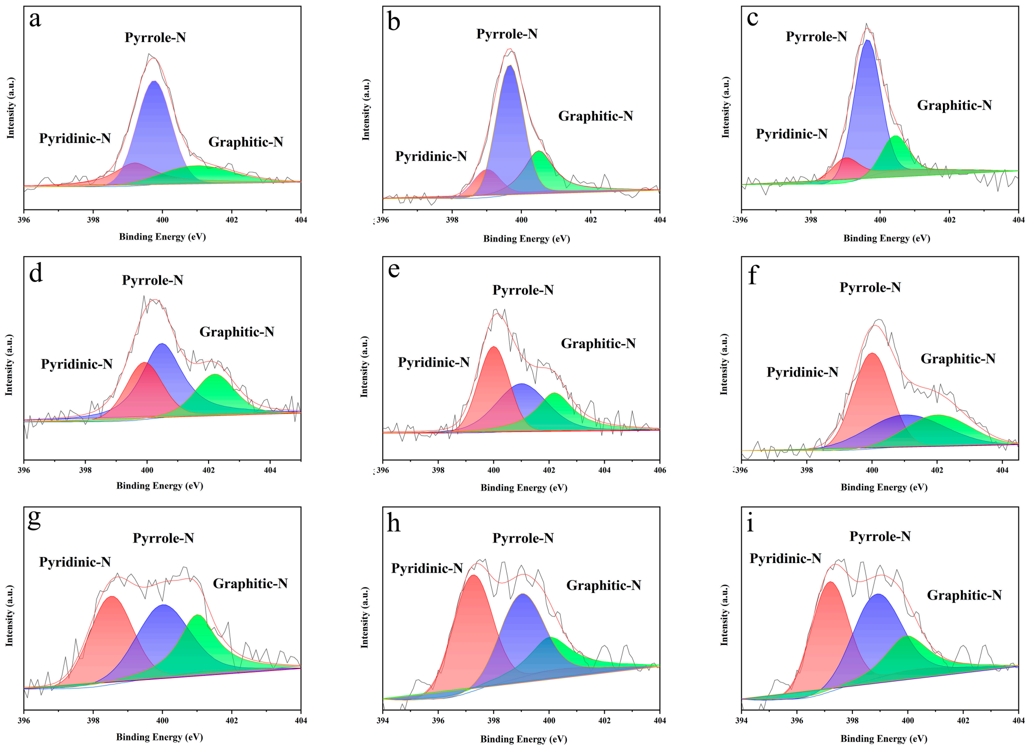

Figure S15. The N1s of the BQH, BQH-AN, BQH-MV adsorbed with RhB (a-c), TC (d-f) and Cr(VI) (g-i).

Reference

[S1] X. Li, J. Shi, X. Luo, Enhanced adsorption of rhodamine B from water by Fe-N co-modified biochar: Preparation, performance, mechanism and reusability, Bioresource Technol., 343 (2022) 126103.

[S2] W. Xiao, Z. N. Garba, S. Sun, I. Lawan, L. Wang, M. Lin, Z. Yuan, Preparation and evaluation of an effective activated carbon from white sugar for the adsorption of rhodamine B dye, J. Clean. Prod., 253 (2020) 119989.

[S3] T. Maneerung, J. Liew, Y. Dai, S. Kawi, C. Chong, C. Wang, Activated carbon derived from carbon residue from biomass gasification and its application for dye adsorption: Kinetics, isotherms and thermodynamic studies, Bioresource Technol., 200 (2016) 350-359.

[S4] S. Wang, B. Yang, Y. Liu, Synthesis of a hierarchical SnS2 nanostructure for efficient adsorption of Rhodamine B dye, J. Colloid Interf. Sci., 507 (2017) 225-233.

[S5] K. Liu, H. Li, Y. Wang, X. Gou, Y. Duan, Adsorption and removal of rhodamine B from aqueous solution by tannic acid functionalized graphene, Colloid. Surf. A, 477 (2015) 35-41.

[S6] Y. Wang, T. Huo, Y. Wang, J. Bai, P. Huang, C. Li, S. Deng, H. Mei, J. Q, X. Zhang, C. Ding, Q. Zhang, W. Wang, Constructing mesoporous biochar derived from waste carton: Improving multi-site adsorption of dye wastewater and investigating mechanism, Environ. Res., 242 (2024) 117775.

[S7] J. Anandkumar, B. Mandal, Adsorption of chromium(VI) and Rhodamine B by surface modified tannery waste: Kinetic, mechanistic and thermodynamic studies, J. Hazard. Mater., 186 (2011) 1088-1096.

[S8] F. Hayeeye, M. Sattar, W. Chinpa, O. Sirichote, Kinetics and thermodynamics of Rhodamine B adsorption by gelatin/activated carbon composite beads, Colloid. Surface. A, 513 (2017) 259-266.

[S9] H. M. H. Gad, A. A. El-Sayed, Activated carbon from agricultural by-products for the removal of Rhodamine-B from aqueous solution, J.Hazard. Mater., 168 (2009) 1070-1081.

[S10] J. Yu, B. Li, X. Sun, J. Yuan, R. Chi, Polymer modified biomass of baker's yeast for enhancement adsorption of methylene blue, rhodamine B and basic magenta, J. Hazard. Mater., 168 (2009) 1147-1154.

[S11] J. Indujalekshmi, M.S. Arsha, V. Biju, KOH-mediated structural modification of activated charcoal by heat treatment for the efficient adsorption of organic dyes, J. Environ. Manage., 206 (2018) 170-177.

[S12] X. Wang, S. Chen, J. Sun, D. Zhang, Z. Yan, X. Xu, Synthesis of large pore sized mesoporous carbon using alumina-templated strategy for high-performance RhB removal, J. Song, Micropor. Mesopor. Mat., 318 (2021) 110993.

[S13] Y. Huang, X. Zheng, S. Feng, Z. Guo, S. Liang, Enhancement of rhodamine B removal by modifying activated carbon developed from Lythrum salicaria L. with pyruvic acid, Colloid. Surface. A, 489 (2016) 154-162.

[S14] T. Zhang, J. Huang, N-vinylimidazole modified hyper-cross-linked resins and their adsorption toward Rhodamine B: Effect of the cross-linking degree, J. Taiwan Inst. Chem. E., 80 (2017) 293-300.

[S15] T. Somsiripan, C. Sangwichien, Enhancement of adsorption capacity of Methylene blue, Malachite green, and Rhodamine B onto KOH activated carbon derived from oil palm empty fruit bunches, Arab. J. Chem., 16 (2023) 105270.

[S16] J. Gong, R. Liu, Y. Sun, J. Xu, M. Liang, Y. Sun, L. Long, Preparation of high-performance nitrogen doped porous carbon from cork biomass by K_2CO_3 activation for adsorption of rhodamine B, Ind. Crop. Prod., 208 (2024) 117846.

[S17] Q. Wang, D. He, C. Li, Z. Sun, J. Mu, Honeycomb-like cork activated carbon modified with carbon dots for high-efficient adsorption of Pb(II) and rhodamine B, Ind. Crop. Prod., 196 (2023) 116485.

[S18] X. Gao, M. Zheng, X. Zhao, S. Song, Z. Gao, Ultra-High-Capacity Adsorption of Rhodamine B in a Carboxyl-Functionalized Metal-Organic Framework via Surface Adsorption, J. Chem. Eng. Data 66 (2021) 669-676.

[S19] Y. Chen, F. Wang, L. Duan, H. Yang, J. Gao, Tetracycline adsorption onto rice husk ash, an agricultural waste: Its kinetic and thermodynamic studies, J. Mol. Liq., 222, (2016) 487-494.

[S20] K.S.D. Premarathna, A.U. Rajapaksha, N. Adassoriya, B. Sarkar, N.M.S. Sirimuthu, A. Cooray, Y.S. Ok, Vithanage, Clay-biochar composites for sorptive removal of tetracycline antibiotic in aqueous media, J. Environ. Manage., 238, (2019) 315-322.

[S21] H. Yu, L. Gu, L. Chen, H. Wen, D. Zhang, H. Tao, Activation of grapefruit derived biochar by its peel extracts and its performance for tetracycline removal, Bioresour. Technol., 316, (2020) 123971.

[S22] H. Huang, Z. Niu, R. Shi, J. Tang, L. Lv, J. Wang, Y. Fan, Thermal oxidation activation of hydrochar for tetracycline adsorption: the role of oxygen concentration and temperature, Bioresour. Technol., 306, (2020) 123096.

[S23] D. Zhang, Q. He, X. Hu, K. Zhang, C. Chen, Y. Xue, Enhanced adsorption for the removal of tetracycline hydrochloride (TC) using ball-milled biochar derived from crayfish shell, Colloid. Surf. A, 615 (2021) 126254.

[S24] W. Xiang, Y. Wan, X. Zhang, Z. Tan, T. Xia, Y. Zheng, B. Gao, Adsorption of tetracycline hydrochloride onto ball-milled biochar: Governing factors and mechanisms, Chemosphere, 255 (2020) 127057.

[S25] A.A. Oladipo, A.O. Ifebajo, Highly efficient magnetic chicken bone biochar for removal of tetracycline and fluorescent dye from wastewater: two-stage adsorber analysis, J. Environ. Manage., 209 (2018) 9-16.

[S26] Y. Zhou, Y. He, Y. He, X. Liu, B. Xu, J. Yu, C. Dai, A. Huang, Y. Pang, L. Luo, Analyses of tetracycline adsorption on alkali-acid modified magnetic biochar: Site energy distribution consideration, Sci. Total Environ., 650 (2019) 2260-2266.

[S27] J.H. Miao, F.H. Wang, Y.J. Chen, Y.Z. Zhu, Y. Zhou, S.T. Zhang, The adsorption performance of tetracyclines on magnetic graphene oxide: a novel antibiotics absorbent, Appl. Surf. Sci., 475 (2019) 549-558.

[S28] Z. Yang, Z. Zhao, X. Yang, Z. Ren, Xanthate modified magnetic activated carbon for efficient removal of cationic dyes and tetracycline hydrochloride from aqueous solutions, Colloid. Surface. A, 615 (2021) 126273.

[S29] Y. Gao, Y. Li, L. Zhang, H. Huang, J. Hu, S.M. Shah, X. Su, Adsorption and

removal of tetracycline antibiotics from aqueous solution by graphene oxide, J. Colloid Interface Sci., 368 (2012) 540-546.

[S30] A. Martins, O. Pezoti, A. Cazetta, K. Bedin, D. Yamazaki, G. Bandoch, T. Asefa, J. Visentainer, V. Almeida, Removal of tetracycline by NaOH-activated carbon producedfrom macadamia nut shells: Kinetic and equilibrium studies, Chem. Eng. J., 260 (2015) 291-299.

[S31] G. Yang, Q. Gao, S. Yang, S. Yin, X. Cai, X. Yu, S. Zhang, Y. Fang, Strong adsorption of tetracycline hydrochloride on magnetic carbon-coated cobalt oxide nanoparticles, Chemosphere, 239 (2020) 124831.

[S32] Y. Chen, J. Liu, Q. Zeng, Z. Liang, X. Ye, Y. Lv, M. Liu, Preparation of Eucommia ulmoides lignin-based high-performance biochar containing sulfonic group: Synergistic pyrolysis mechanism and tetracycline hydrochloride adsorption, Bioresour. Technol., 329 (2021) 124856.

[S33] S. Masoumi, F.F. Tabrizi, A.R. Sardarian, Efficient tetracycline hydrochloride removal by encapsulated phosphotungstic acid (PTA) in MIL-53 (Fe): Optimizing the content of PTA and recycling study, J. Environ. Chem. Eng. 8 (2020) 103601.

[S34] H.M. Perera, A.U. Rajapaksha, S. Liyanage, A. Ekanayake, R. Selvasembian, A. Daverey, M. Vithanage, Enhanced adsorptive removal of hexavalent chromium in aqueous media using chitosan-modified biochar: Synthesis, sorption mechanism, and reusability, Environ. Res. 231 (2023) 115982.

[S35] F. Xiao, J. Cheng, W. Cao, C. Yang, J. Chen, Z. Luo, Removal of heavy metals from aqueous solution using chitosan-combined magnetic biochars, J. Colloid. Interface. Sci. 540 (2019) 579–584.

[S36] T. Altun, H. Ecevit, Y. Kar, B. Çiftçi, Adsorption of Cr(VI) onto cross-linked chitosan-almond shell biochars: equilibrium, kinetic, and thermodynamic studies, J. Anal. Sci. Technol. 12 (2021) 38.

[S37] L. Luo, S. Cheng, L. Yue, Z. You, J. Cai, N-doped biochar from chitosan gel-like solution: Effect of hydrothermal temperature and superior aqueous Cr (VI) removal performance, Colloids. Surf. A Physicochem. Eng. Asp. 641 (2022)

128426.
[S38] Y. Yang, Y. Zhang, G. Wang, Z. Yang, J. Xian, Y. Yang, T. Li, Y. Pu, Y. Jia, Y. Li, Z. Cheng, S. Zhang, X. Xu, Adsorption and reduction of Cr(VI) by a novel nanoscale FeS/chitosan/biochar composite from aqueous solution, J. Environ. Chem. Eng. 9 (2021) 105407.
[S39] B. Wang, Y. Zeng, M. Xiong, R. Qiu, Adsorption performance and mechanism of mesoporous carbon-doped Al_2O_3 adsorbent derived from NH_2-MIL-53 (Al) for removing Cr(VI) and methyl orange from aqueous solution, J. Environ. Chem. Eng. 11 (2023) 110081.
[S40] H. Wang, W. Wang, S. Zhou, X. Gao, Adsorption mechanism of Cr(VI) on woody-activated carbons, Heliyon 9 (2023) e13267.
[S41] S.M. Kharrazi, M. Soleimani, M. Jokar, T. Richards, A. Pettersson, N. Mirghaffari, Pretreatment of lignocellulosic waste as a precursor for synthesis of high porous activated carbon and its application for Pb (II) and Cr (VI) adsorption from aqueous solutions, Int. J. Biol. Macromol. 180 (2021) 299-310.
[S42] H. Xu, Y. Liu, H. Liang, C. Gao, J. Qin, L. You, R. Wang, J. Li, S. Yang, Adsorption of Cr(VI) from aqueous solutions using novel activated carbon spheres derived from glucose and sodium dodecylbenzene sulfonate, Sci. Total Environ. 759 (2021) 143457.
[S43] X. Chen, Z. Song, B. Yuan, X. Li, S. Li, T.T. Nguyen, M. Guo, Z. Guo, Fluorescent carbon dots crosslinked cellulose Nanofibril/Chitosan interpenetrating hydrogel system for sensitive detection and efficient adsorption of Cu (II) and Cr (VI), Chem. Eng. J. 430 (2022) 133154.
[S44] H. Liang, K. Ma, X. Zhao, Z. Geng, D. She, H. Hu, Enhancement of Cr(VI) adsorption on lignin-based carbon materials by a two-step hydrothermal strategy: Performance and mechanism, Int. J. Biol. Macromol. 252 (2023) 126432.
[S45] X. Yang, B. Wang, P. Zhang, X. Song, Adsorption and reduction of Cr(VI) by N, S co-doped porous carbon from sewage sludge and low-rank coal: Combining experiments and theoretical calculations, Sci. Total Environ. 912 (2024) 169265.
